# Functional and Cognitive Decline Is Associated With Increased Endothelial Cell Inflammation and Platelet Activation: Liquid Biopsy of Microvesicles in Community- Dwelling Octogenarians

**DOI:** 10.3389/fcell.2021.716435

**Published:** 2021-07-29

**Authors:** Gemma Chiva-Blanch, Alba Vilella-Figuerola, Teresa Padró, Francesc Formiga, Assumpta Ferrer, Lina Badimon

**Affiliations:** ^1^Cardiovascular Program ICCC, Institut de Recerca Hospital Santa Creu i Sant Pau-IIB Sant Pau, Barcelona, Spain; ^2^Endocrinology and Nutrition Department, Institut d’Investigacions Biomèdiques August Pi Sunyer, Hospital Clínic, Barcelona, Spain; ^3^Centro de Investigación Biomédica en Red Fisiopatología de la Obesidad y Nutrición, Instituto de Salud Carlos III, Madrid, Spain; ^4^Centro de Investigación Biomédica en Red Cardiovascular, Instituto de Salud Carlos III, Madrid, Spain; ^5^Geriatric Unit, Internal Medicine Service, Hospital Universitari de Bellvitge, Barcelona, Spain; ^6^Bellvitge Biomedical Research Institute, IDIBELL, L’Hospitalet de Llobregat, Barcelona, Spain; ^7^Primary Healthcare Centre “El Plà” CAP-I, Sant Feliu de Llobregat, Barcelona, Spain

**Keywords:** circulating microvesicles, octogenarians, successful aging, cognitive decline, inflammation, platelet activation, thrombomodulin, endothelial cells

## Abstract

Increased life expectancy is usually associated with comorbidities, such as cardio and cerebrovascular disease causing impaired functionality. A common underlying cause of these comorbidities is vascular inflammation and injury. Elevated levels of circulating microvesicles (cMV), as a product of a hemostatic and inflammatory cell activation, could be direct mapping of an imbalanced hemostasis. In this manuscript, we aimed to investigate by liquid biopsy whether successful aging can be discriminated by cMV levels and phenotype. To this purpose, we included 135 community-dwelling octogenarians in a cross-sectional study. Successful aging was defined as good functional (Barthel Index > 90 points, and Lawton index score > 7/4 points for women and men, respectively) and cognitive status (Spanish version of the Mini-Mental State Examination -MEC- > 24 points) and no need for institutionalization. Total, annexin V positive (AV^+^), and AV^–^ cMV from different cell origins from the vascular compartment were phenotypically characterized and quantified from fasting plasma samples by flow cytometry. Successful aging was associated with lower plasma concentrations of total and AV^+^ CD141^+^/CD41^+^-CD61^+^, and PAC1^+^/AV^+^, CD141^+^/AV^+^, and CD36^+^/AV^–^ cMV. From these phenotypes, ROC curve analyses revealed that CD141^+^/AV^+^ and CD141^+^/CD41^+^-CD61^+^/AV^+^ endothelial- and platelet-derived cMV discriminate successful and non-successful aging with an AUC (95%CI) of 0.655 (0.551, 0.758), *P* = 0.005, and 0.638 (0.535, 0.741), *P* = 0.013, respectively. In conclusion, successful aging is associated with low levels of cMV released by endothelial cells and platelets, indicating lower endothelial cell inflammation and platelet activation. Our results contribute to the understanding of the link between unsuccessful aging, cognitive decline and vascular cell inflammatory disturbances.

## Introduction

Successful aging can be defined as growing old in optimum conditions (von Faber et al., 2001), considering the fact that increased life expectancy is usually associated with comorbidities, impaired functionality (Mitra et al., 2020), and diminished quality of life (Formiga et al., 2011). The 2018 Aging Report from the European Union confirms that the European population is continuously aging in a significant manner (European Commission [EC], 2018). Although life expectancy may be currently affected by the COVID pandemic (Trias-Llimós and Bilal, 2020), hopefully in a passing manner, the old age-related dependency is expected to increase in more than 20% in 2070 (European Commission [EC], 2018).

Non-successful aging is associated with higher risk of malnutrition and falls, frailty, and with higher co-morbidity, and more specifically with cardiovascular disease (CVD) and cerebrovascular disease (Formiga et al., 2011; Beauchet et al., 2020), with vascular damage as a common underlying cause. In fact, it is of paramount importance to reduce the incidence of major vascular events such as coronary heart disease or stroke, in order to prevent cognitive and functional decline, or even disability.

Circulating microvesicles (cMV) are membrane blebs ranged 0.1–1 μm extruded from almost all types of cells after activation, injury or apoptosis. cMV contain bioactive molecules that can modulate the phenotype of target recipient cells by paracrine regulation (Gnecchi et al., 2006; Badimon et al., 2019). Indeed, cMV can bind and activate endothelial cells, that by recruiting platelets and leukocytes and inducing smooth muscle cell proliferation, may overall enhance vascular inflammation and damage (Daniel et al., 2006; Dalli et al., 2013; Hulsmans and Holvoet, 2013). Elevated concentrations of cMV have been observed after a stroke (Chiva-Blanch et al., 2016) or coronary heart disease (Chiva-Blanch et al., 2017a), and are believed to be both the cause and consequence of atherosclerosis. We have recently observed that frail older adults also show elevated levels of cMV from platelets and leukocytes (Arauna et al., 2020). Therefore, specific cMV phenotypes from blood and vascular cells associated with non-successful aging, may be candidate biomarkers of age-related cognitive and functional impairment. These may help in the research of prognostic biomarkers indicators of overall functional and cognitive decline in the future, and may help in the identification of subjects requiring specific strategies to prevent vascular dysfunction at the onset of cognitive and functional decline.

Previously, we observed that older octogenarians with non-successful aging had altered levels in plasma of proteins related to inflammation and coagulation, which were directly associated with cognitive status, cardiovascular event presentation (Cubedo et al., 2015) and increased cardiovascular mortality at 5 years follow-up (Cubedo et al., 2017). Considering the biological link between aging, vascular damage, cognitive decline and functional performance, and the worldwide importance of successful aging in the society, the aim of our study is to investigate whether successful aging is related to a lower activation of cells in the vascular compartment through the quantification of their MV release.

## Materials and Methods

### Subjects

In this cross-sectional study, we included 135 community-dwelling octogenarians from the OCTABAIX (Oldest CharacTheristic and Assessments-Baix Llobregat) cohort (Ferrer et al., 2010; Badia et al., 2015) at baseline (registered at clinicaltrials.gov with the reference NCT01141166). Inclusion criteria, as per protocol, were to be born in 1924 and not being institutionalized (24 h of professional care available). Therefore, all subjects were 85 years old at the year of inclusion (2009). Exclusion criteria in this sub-study was having a cardiovascular event within the last year. In addition, samples with repeated freeze/thaw cycles and hemolyzed samples were also excluded (Supplementary Figure 1). All samples were processed within October-November 2019.

Functional, cognitive and nutritional status were assessed with questionnaires currently used in geriatric practice. Barthel Index was performed to measure the functional capacity for basic activities of daily living; Lawton Index was administered to evaluate the ability to carry out instrumental activities; and the Spanish version of the Mini-Mental State Examination (MEC) was performed to assess cognitive impairment. In the three test, the higher the score, the higher the functionality and independence. Subjects with Barthel Index scores > 90 were classified as functional independent. Women with Lawton Index scores > 7 and men with scores > 4, respectively, (Graf, 2008), were considered able for instrumental activities, and subjects above 24 points in the MEC score showed minimal cognitive impairment. Nutritional status was evaluated with the mini-nutritional assessment (MNA), and scores below 23.5 were classified as malnutrition or at risk. Successful aging was defined as good functional (Barthel Index > 90 points, and Lawton Index scores > 7 for women and >4 men) and cognitive status (MEC > 24 points), and no need for institutionalization (Formiga et al., 2011). Non-successful aging was further categorized in having one, two or three scores below good functional and/or cognitive status (Barthel Index ≤ 90 points, Lawton Index scores ≤ 7 for women and ≤4 men, and/or MEC ≤ 24 points). Medical data were primarily obtained from general practice records, and interviews were performed at primary care services. Subjects signed an informed consent before inclusion in the study, and all procedures were conducted in accordance with the Declaration of Helsinki.

### Blood Sampling

Fasting venous blood was withdrawn using a 20-gage needle, and blood was collected into EDTA-treated tubes. Blood cells were removed by centrifugation (1,500 × g, 15 min) at room temperature (RT). Plasma was carefully aspirated, and 250 μL aliquots of EDTA-anticoagulated plasma were immediately frozen and stored at −80∘C until processing.

### Laboratory Measurements

Biochemical and hematological parameters were quantified by standardized methods.

### Isolation, Phenotyping and Quantification of Circulating Microvesicles

Circulating microvesicles were quantified as previously (Chiva-Blanch et al., 2019, 2020; Arauna et al., 2020) and currently described in Supplementary Material. Briefly, cMV were isolated from 250 μL plasma by differential centrifugation and were triple-label stained with Annexin V (AV, which has high affinity for phosphatidylserine) and two specific monoclonal antibodies (Supplementary Table 1) labeled with fluorescein isothiocyanate (FITC) or phycoerythrin (PE), prior flow cytometric analyses in a FACSCanto II (BD Biosciences). Flow cytometry controls are shown in Supplementary Figure 2. cMV were phenotypically characterized for bioactive and/or biomarker molecules from their parental cells and were defined as total cMV (AV^±^), AV^+^, or AV^–^, according to phosphatidylserine exposure (Supplementary Figure 3). Phosphatidylserine exposure promotes thrombin generation and thrombus formation. Therefore, AV^+^ cMV present higher prothrombotic activity than platelets (Sinauridze et al., 2007).

### Statistical Analyses

Sample size was determined with the ENE 3.0 statistical program (GlaxoSmithKline, Brentford, United Kingdom) assuming a loss of 0% participants because samples were already collected. We estimated that to recognize as statistically significant a difference in the number of PAC-1^+^/AV^+^ cMV of 5 units (MV)/μL plasma with a conservative SD of 6, we needed 19 and 28 subjects per group (successful and non-successful aging), accepting an alpha risk of 0.05 and a beta risk of 0.2 in a two-sided test, and considering the proportion of successful aging around 0.30 of the population (Formiga et al., 2011). Although PAC-1^+^/AV^+^ was used to determine sample size, all cMV phenotypes tested were considered primary outcomes. Therefore, to ensure sufficient power for the other outcomes and to ensure sufficient statistical power to control for potential confounders, the sample size was nearly doubled according to sample availability (Supplementary Figure 1).

Statistical analyses were performed using the SPSS Statistical Analysis System (version 23.0). Results are expressed as mean ± SD, mean + SEM, or *n* (%) when indicated. Normality of variables was assessed with the Shapiro–Wilk test, and all variables followed a non-parametric distribution. Therefore, non-parametric analyses were conducted. To analyze differences according to functional and cognitive status or successful aging, Mann–Whitney test for two independent samples (for the comparison between successful and non-successful aging), or the Kruskal–Wallis test for K independent samples (for the comparison between different degrees of non-successful aging) were carried out. Multiple comparisons were performed with the Bonferrroni *post hoc* test. Multivariable models for successful aging were performed with logistic regression models (conventional and stepwise) with cMV from different cell origins and phenotypes adjusting by potential confounders in two different models. The first model, was adjusted by previous CVD (yes/no), psychotropics (yes/no) and MNA > 23.5, according to stepwise regression. The second model was further adjusted by sex, dyslipidemia (yes/no), statins (yes/no), and antiplatelet agents (yes/no), factors known to modulate MV release (Badimon et al., 2019), and with existing significant differences between groups. Receiver operating characteristic (ROC)-curve estimations were performed, and their corresponding C statistics [area under the curve (AUC) with their 95% CI] were calculated for cMV phenotypes associated with successful aging. A 2-tailed *P* value of <0.05 was considered statistically significant.

## Results

### Subjects Characteristics

Table 1 depicts the clinical characteristics of the subjects according to their aging success. Subjects with a successful aging were women in a lower percentage, showed higher levels of HDL cholesterol and the renal function was more preserved than in non-healthy aged subjects. No differences in the levels of circulating leukocytes, platelets, hemoglobin, transaminases, albumin, total protein, thyroid stimulating hormone or tiroxin 4 were observed between subjects with and without successful aging (not shown).

**TABLE 1:** Characteristics of the 85-year old subjects included in the study according to successful aging.

	**Non-successful aging (*n* = 87)**			**Successful aging (*n* = 48)**	***P*^1^**	***P*^2^**
	**First degree (*n* = 28)**	**Second degree (*n* = 23)**	**Third degree (*n* = 36)**			
Females [*n* (%)]	22 (78.6)	18 (78.3)	23 (63.9)	24 (50.0)	0.009	0.033
Systolic blood pressure (mmHg)	136.68 ± 16.57	137.52 ± 16.59	135.14 ± 20.74	137.56 ± 16.68	0.591	0.932
Diastolic blood pressure (mmHg)	72.43 ± 10.84	71.04 ± 9.33	70.64 ± 11	71.4 ± 11.17	0.874	0.938
BMI (Kg/m^2^)	28.76 ± 5.48	28.22 ± 4.83	29.85 ± 3.99	28.58 ± 4.12	0.226	0.498
Glucose (mmol/L)	5.37 ± 1.48	5.56 ± 1.85	5.81 ± 1.82	5.83 ± 0.98	0.498	0.567
HbA1C (%)	6.35 ± 1.58	6.01 ± 1.06	6.08 ± 0.91	5.89 ± 0.56	0.393	0.303
Triglycerides (mmol/L)	1.35 ± 0.56	1.56 ± 0.64	1.32 ± 0.57	1.24 ± 0.6	0.056	0.216
Total cholesterol (mmol/L)	5.12 ± 1.14	5.09 ± 1.26	4.66 ± 0.75	5.24 ± 0.85	0.069	0.053
HDL cholesterol (mmol/L)	1.48 ± 0.34	1.32 ± 0.4	1.39 ± 0.3	1.62 ± 0.45	0.002	0.009
LDL cholesterol (mmol/L)	3.03 ± 1.03	3.08 ± 1.17	2.67 ± 0.67	3.06 ± 0.73	0.218	0.165
Tobacco consumption [*n* (%)]					0.151	0.183
Current smokers	1 (3.6)	1 (4.34)	0 (0)	0 (0)		
Former smokers	3 (10.7)	3 (13.0)	10 (27.8)	15 (31.2)		
Creatinin (mg/dL)	77.82 ± 19.26	100.35 ± 62.2	101.86 ± 31.45	82.75 ± 20.91	0.019	0.008
Glomerular filtration (mL/min)	57.42 ± 7.02	52.94 ± 14.6	51.04 ± 10.37	58.22 ± 5.72	0.005	0.002
**Functional, cognitive and nutritional indexes**						
BARTHEL	93.04 ± 6.43	72.61 ± 24.58	62.36 ± 27.03	98.54 ± 2.3	<0.0001	<0.0001
BARTHEL > 90	16 (57.1)	2 (8.7)	0 (0)	48 (100)	<0.0001	<0.0001
LAWTON	6.07 ± 1.72	3.78 ± 2.13	1.89 ± 1.7	6.9 ± 1.37	<0.0001	<0.0001
LAWTON > 7 (women)	14 (63.4)	3 (16.7)	0 (0)	24 (100)	<0.0001	<0.0001
LAWTON > 4 (men)	4 (66.7)	0 (0)	0 (0)	24 (100)	<0.0001	<0.0001
MEC	28.18 ± 4.78	26.3 ± 5.47	16.08 ± 5.49	31.29 ± 2.76	<0.0001	<0.0001
MEC > 24	22 (78.6)	18 (78.2)	0 (0)	48 (100)	<0.0001	<0.0001
MNA	25.38 ± 1.88	20.78 ± 4.04	22.29 ± 2.9	26.74 ± 2.95	<0.0001	<0.0001
MNA > 23.5	24 (85.7)	7 (30.4)	13 (36.1)	43 (89.6)	<0.0001	<0.0001
Dyslipidemia [*n* (%)]	13 (46.4)	11 (47.8)	14 (38.9)	8 (16.7)	0.002	0.014
Diabetes [*n* (%)]	10 (35.7)	6 (26.1)	19 (52.8)	12 (25.0)	0.126	0.075
Years of evolution	13 ± 8.33	16 ± 8.29	13.89 ± 5.47	8.58 ± 4.4	0.027	0.075
History of CVD [*n* (%)]	11 (39.3)	15 (65.2)	32 (88.9)	12 (25.0)	<0.0001	<0.0001
Myocardial infarction	3 (10.7)	5 (21.7)	10 (27.8)	6 (12.5)	0.234	0.205
Heart failure	4 (14.3)	12 (52.2)	8 (22.2)	2 (4.2)	0.001	<0.0001
Peripheral vascular disease	1 (3.6)	1 (4.3)	5 (13.9)	1 (2.1)	0.16	0.125
Stroke	4 (14.3)	9 (39.1)	16 (44.4)	4 (8.3)	0.001	<0.0001
Atrial fibrillation	4 (14.3)	5 (21.7)	7 (19.4)	3 (6.25)	0.052	0.221
Chronic renal failure [*n* (%)]	0 (0)	3 (13.0)	3 (8.3)	3 (6.25)	0.885	0.298
**Medication [*n* (%)]**						
Psychotropic	9 (32.1)	16 (69.6)	21 (58.3)	12 (25.0)	0.002	<0.0001
Cardiac dysrhythmia medication	3 (10.7)	5 (21.7)	6 (16.7)	6 (12.5)	0.574	0.671
Statins	14 (50)	10 (43.5)	18 (50)	11 (22.9)	0.004	0.034
ACE Inhibitors	16 (57.1)	10 (43.5)	17 (47.2)	14 (29.2)	0.023	0.098
Angiotensin-II receptor blockers	2 (7.1)	4 (17.4)	14 (38.9)	8 (16.7)	0.386	0.012
β- Blockers	8 (28.6)	5 (21.7)	11 (30.6)	6 (12.5)	0.044	0.193
α- Blockers	0 (0)	0 (0)	8 (22.2)	7 (14.6)	0.34	0.009
Calcium channel blockers	4 (14.3)	8 (34.8)	14 (38.9)	6 (12.5)	0.023	0.013
Diuretics	18 (64.3)	16 (69.6)	22 (61.1)	22 (45.8)	0.037	0.19
Antiplatelet agents	10 (35.7)	9 (39.1)	21 (58.3)	9 (18.7)	0.009	0.003
Oral anticoagulant therapy	3 (10.7)	4 (17.4)	8 (22.2)	4 (8.3)	0.154	0.289
Oral Antidiabetic agents	8 (28.6)	4 (17.4)	8 (22.2)	7 (14.6)	0.243	0.498
Insulin	2 (7.1)	4 (17.4)	4 (11.1)	0 (0)	0.015	0.048

*P^1^ for the comparison between successful and non-successful aging (Mann–Whitney test or Chi-squared test), and P^2^, for the comparison between successful aging and different degrees of non-successful aging (Kruskal–Wallis test or Chi-squared test). Successful aging was defined as good functional (Barthel Index > 90 points, and Lawton Index scores > 7 for women and >4 men) and cognitive status (MEC > 24 points), and no need for institutionalization. Non-successful aging was further categorized in having one (first degree), two (second degree), or three scores (third degree) below good functional and/or cognitive status (Barthel Index ≤ 90 points, Lawton Index scores ≤ 7 for women and ≤4 men, and/or MEC ≤ 24 points). Glomerular filtration was calculated with the Modification of Diet in Renal Disease formula (MDRD). BMI indicates body mass index; HDL, high density lipoprotein; LDL, low density lipoprotein; MNA, mini-nutritional assessment; MEC, Spanish version of the Mini-Mental State Examination; CVD, cardiovascular disease; and ACE, angiotensin-converting-enzyme.*

As expected, and as per definition, subjects with a successful aging scored higher in the Barthel and Lawton indexes, and MEC and MNA scores compared to non-successfully aged subjects. Consequently, a higher proportion of subjects with successful aging were classified as functionally independent (Barthel Index > 90), able to conduct instrumental activities (Lawton Index > 7 or 4 for women and men, respectively), with high cognitive performance (MEC score > 24), and/or adequately nourished (MNA scores ≥ 24).

Subjects who reported non-successful aging had a higher incidence of previous CVD (heart failure and stroke). Consequently, these subjects were in more proportion under statins, β- blockers, calcium channel blockers, and antiplatelet therapies. In addition, a higher percentage of subjects who reported non-successful aging were under psychotropic medication.

### MV- Functional, Cognitive and Nutritional Status

Subjects with a Barthel index score > 90 (*n* = 66), indicating functional independence, showed lower levels of platelet-, endothelial-, immune cell-, and erythrocyte- derived total, AV^+^ or AV^–^ cMV compared to dependent subjects (Barthel score ≤ 90, *n* = 69; *P* ≤ 0.049, all), as can be observed in Supplementary Figure 4.

Individuals with a Lawton index score > 7 for women and >4 for men (*n* = 69), indicating ability for instrumental activities, presented lower levels of platelet-derived total and AV^+^ GPVI^+^ and CD36^+^/GPVI^+^ cMV compared to subjects with a significant degree of disability (*n* = 66), as illustrated in Supplementary Figure 5 (*P* ≤ 0.042, all).

In the same line, octogenarians with a MEC score < 24 (*n* = 47), indicating cognitive impairment, showed higher concentrations of platelet-derived total and AV^+^ CD36^+^/GPVI^+^ and GPVI^+^/AV^+^ cMV; and CD9^+^/AV^–^ and CD36^+^/AV^–^ cMV compared to subjects with MEC scores ≥ 24 (*n* = 88), as depicted in Supplementary Figure 6 (*P* ≤ 0.034, all).

Supplementary Figure 7 shows that subjects with or at risk of malnutrition (MNA ≤ 23.5; *n* = 48) show higher concentrations of platelet-derived total, AV^+^ and AV^–^ cMV compared to subjects with MNA scores ≥ 24 (*n* = 87), *P* ≤ 0.047, all, indicating platelet hyperreactivity in this situation. On the other hand, subjects with MNA ≤ 23.5 showed lower concentrations of immune cell-derived total CD9^+^ and CD9^+^/AV^+^, and higher concentrations of CD40^+^/AV^+^ and CD16^+^/AV^–^ cMV compared to subjects adequately nourished (*P* ≤ 0.043, all), suggesting altered immune functionality in subjects with malnutrition.

No significant associations were found between all other MV phenotypes determined and functional, cognitive and nutritional status.

### MV- Successful Aging

**FIGURE 1:**
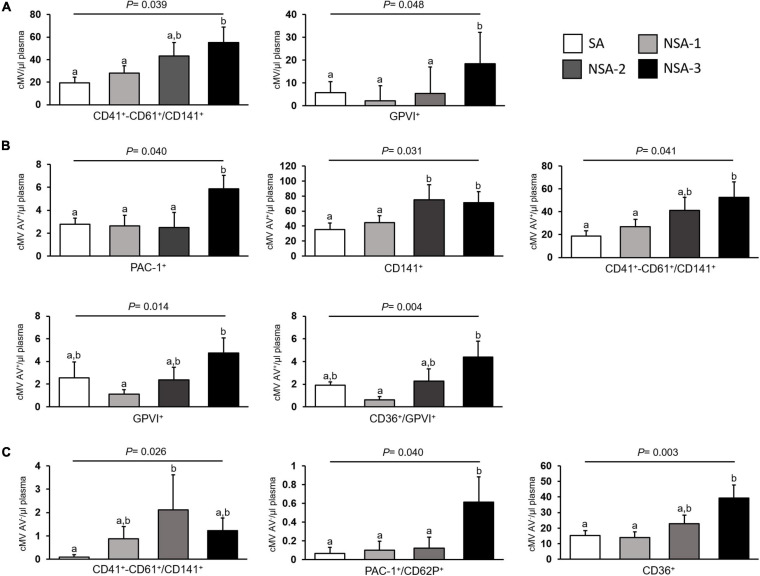
cMV levels according to successful aging in the 135 octogenarians included in the study. Results are shown as mean + SEM of concentrations of: **(A)** total microvesicles; **(B)** AV^+^ microvesicles; and **(C)** AV^–^ microvesicles. Successful aging (*n* = 48) was defined as good functional (Barthel Index > 90 points, and Lawton Index scores > 7 for women and >4 men) and cognitive status (MEC > 24 points), and no need for institutionalization. Non-successful aging (NSA, *n* = 87) was further categorized in having one (NSA-1, *n* = 28), two (NSA-2, *n* = 23), or three scores (NSA-3, *n* = 36) below good functional and/or cognitive status (Barthel Index ≤ 90 points, Lawton Index scores ≤ 7 for women and ≤4 men, and/or MEC ≤ 24 points). AV indicates annexin V and cMV, circulating microvesicles. Used markers for MV phenotyping are shown in Supplementary Table 1. Columns with different superscript letters indicate significant differences (P < 0.05, Bonferroni *post hoc* test).

Endothelial-derived CD31^+^/CD41a^–^ (*P* = 0.044), and CD31^+^/CD41a^–^/AV^–^ (*P* = 0.028); platelet-derived CD141^+^ /CD41^+^-CD61^+^ (*P* = 0.009), CD141^+^/CD41^+^-CD61^+^/AV^–^ (*P* = 0.004), CD36^+^/GPVI^+^ (*P* = 0.024), and P2RY12^+^ (*P* = 0.032); as well as CD141^+^ (*P* = 0.033), CD36^+^/AV^–^ (*P* = 0.029), and CD40^+^ (*P* = 0.004) cMV concentrations were lower in subjects who reported a successful aging. Moreover, AV^+^ cMV from platelet origin [CD41^+^-CD61^+^/CD141^+^/AV^+^ (*P* = 0.009) and CD36^+^/GPVI^+^/AV^+^ (*P* = 0.045)], and CD141^+^/AV^+^ (*P* = 0.006) cMV, were also lower in subjects who reported a successful aging, compared to those subjects non-successfully aged. As depicted in Figure 1, when non-successful aging was divided in three degrees of severity, increasing concentrations of total (Figure 1A), AV^+^ (Figure 1B), and AV^–^ (Figure 1C) cMV from platelet origin are observed, according to the severity of cognitive and functional impairment.

**FIGURE 2:**
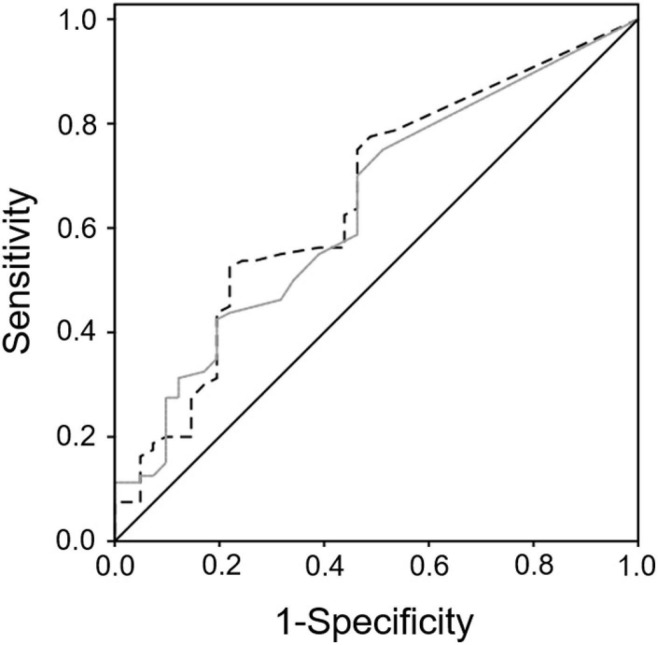
ROC Receiver operating characteristic (ROC)-Curve analysis for successful aging discrimination. ROC curve analyses revealed that CD141^+^/AV^+^ (discontinued black line), and CD141^+^/CD41^+^-CD61^+^/AV^+^ (gray line) endothelial- and platelet-derived circulating microvesicles (cMV) discriminate successful and non-successful aging with an AUC (95%CI) of 0.655 (0.551, 0.758), *P* = 0.005, and 0.638 (0.535, 0.741) *P* = 0.013, respectively. Black continuous line is the reference line of the ROC curve (AUC = 0.5). AV indicates annexin V. Used markers for MV phenotyping are shown in Supplementary Table 1.

As shown in Table 2, after multivariate-adjustments by sex, dyslipidemia, previous CVD, psychotropics, statins, antiplatelet agents, and MNA > 23.5, total and AV^+^ CD141^+^/CD41^+^-CD61^+^, and PAC1^+^/AV^+^, CD141^+^/AV^+^, and CD36^+^/AV^–^ cMV remained inversely associated with successful aging. From these phenotypes, ROC curve analyses revealed that CD141^+^/AV^+^ and CD141^+^/CD41^+^-CD61^+^/AV^+^ endothelial- and platelet-derived cMV modestly but significantly discriminate successful and non-successful aging with an AUC (95%CI) of 0.655 (0.551, 0.758), *P* = 0.005, and 0.638 (0.535, 0.741) *P* = 0.013, respectively, (Figure 2), considering non-successful aging as the state of the variable. However, when both phenotypes were considered together in the ROC analyses, there was no significant improvement for the discrimination between successful and non-successful aging AUC (95%CI): 0.656 (0.554, 0.759), *P* = 0.005. No associations were found between successful aging and all other MV phenotypes determined (Supplementary Table 2).

**TABLE 2:** Association between cMV from different phenotypes and successful aging.

	***B* (95% CI)**	***P***
**Total cMV**		
**CD141^+^/CD41^+^-CD61^+^**		
Unadjusted	0.006 (0002, 0.010)	0.003
Multivariate-adjusted^1^	0.003 (0.000, 0.007)	0.038
Multivariate-adjusted^2^	0.003 (0.000, 0.007)	0.039
**GPVI^+^**		
Unadjusted	0.008 (0.000, 0.016)	0.056
Multivariate-adjusted^1^	Not included in the stepwise model	
Multivariate-adjusted^2^	0.006 (0.000, 0.012)	0.088
**AV^+^ cMV**		
**PAC-1^+^/AV^+^**		
Unadjusted	0.048 (0.009, 0.086)	0.016
Multivariate-adjusted^1^	0.044 (0.014, 0.073)	0.004
Multivariate-adjusted^2^	0.046 (0.017, 0.076)	0.002
**CD141^+^/AV^+^**		
Unadjusted	0.004 (0.001, 0.007)	0.014
Multivariate-adjusted^1^	0.002 (0.000, 0.005)	0.035
Multivariate-adjusted^2^	0.002 (0.000, 0.005)	0.047
**CD141^+^/CD41^+^-CD61^+^/AV^+^**		
Unadjusted	0.006 (0.002, 0.010)	0.003
Multivariate-adjusted^1^	0.003 (0.000, 0.007)	0.045
Multivariate-adjusted^2^	0.003 (0.000, 0.007)	0.043
**GPVI^+^/AV^+^**		
Unadjusted	0.020 (−0.010, 0.051)	0.191
Multivariate-adjusted^1^	Not included in the stepwise model	
Multivariate-adjusted^2^	0.020 (−0.005, 0.045)	0.109
**CD36^+^/GPVI^+^/AV^+^**		
Unadjusted	0.025 (−0.006, 0.056)	0.114
Multivariate-adjusted^1^	Not included in the stepwise model	
Multivariate-adjusted^2^	0.019 (−0.006, 0.043)	0.129
**AV^–^ cMV**		
**CD141^+^/CD41^+^-CD61^+^/AV^–^**		
Unadjusted	0.056 (−0.005, 0.117)	0.071
Multivariate-adjusted^1^	Not included in the stepwise model	
Multivariate-adjusted^2^	0.022 (−0.027, 0.071)	0.378
**PAC-1^+^/CD62P^+^/AV^–^**		
Unadjusted	0.307 (0.064, 0.549)	0.014
Multivariate-adjusted^1^	Not included in the stepwise model	
Multivariate-adjusted^2^	0.195 (0.000, 0.389)	0.050
**CD36^+^/AV^–^**		
Unadjusted	0.01 (0.005, 0.017)	0.001
Multivariate-adjusted^1^	0.011 (0.006, 0.015)	<0.0001
Multivariate-adjusted^2^	0.011 (0.006, 0.016)	<0.0001

*Results are expressed as *B* coefficient of regression with its 95% confidence interval [*B* (95% CI)]. *P* from the regression model unadjusted, and ^1^adjusted by previous CVD (yes/no), psychotropics (yes/no) and MNA > 23.5, according to stepwise regression; and ^2^adjusted by sex, previous CVD (yes/no), psychotropics (yes/no) and MNA > 23.5, dyslipidemia (yes/no), statins (yes/no), and antiplatelet agents (yes/no). AV indicates annexin V and cMV, circulating microvesicles. Used markers for MV phenotyping are shown in Supplementary Table 1. Successful aging was defined as good functional (Barthel Index > 90 points, and Lawton Index scores > 7 for women and >4 men) and cognitive status (MEC > 24 points), and no need for institutionalization. Non-successful aging was categorized in having one, two or three scores below good functional and/or cognitive status (Barthel Index ≤ 90 points, Lawton Index scores ≤ 7 for women and ≤4 men, and/or MEC ≤ 24 points).*

## Discussion

In this cross-sectional study, we have reported an inverse association between successful aging and elevated concentrations of cMV from platelets and endothelial cells in dwelling-community octogenarians. To our knowledge, this is the first time that the link between successful aging and cell activation, through the quantification of cMV release at the bloodstream, has been reported.

Elevated concentrations of cMV from platelets and endothelial cells have been found in subjects after a major cardiovascular event such as an acute myocardial infarction (Chiva-Blanch et al., 2017b, 2019) or stroke (Lee et al., 2012; Chiva-Blanch et al., 2016). These major adverse cardiovascular events are known to cause an important decline in functional and cognitive performance, or even disability because of the lack of oxygen supply. In fact, and as expected, subjects with previous CVD had significantly lower functional, cognitive and nutritional scores compared to subjects free of major adverse cardiovascular events at 85 years old (data not shown). Vascular dysfunction is closely associated with dementia, especially in the oldest (van der Flier et al., 2018). In fact, cerebral amyloid angiopathy, large and lacunar infarcts, micro and large hemorrhages, microaneurysms, perivascular space dilation, and arteriosclerosis predict vascular cognitive impairment (Skrobot et al., 2016), and such situations have also been linked to increased MV release from platelets, endothelial cells and leukocytes (Badimon et al., 2019; Castillo et al., 2019). Given the interrelationship between CVD and cMV, and CVD and functional and cognitive performance, it seems biologically plausible that lower scores of functional and cognitive tests might be associated with high levels of cMV. However, if elevated cMV are the result of cognitive dysfunction (vascular cognitive impairment), or one of its causes still remains unknown.

We have observed that subjects with low functional and cognitive performance, quantified by Barthel and Lawton Indexes and MEC score, show increased levels of cMV with prothrombotic phenotype (AV^+^) derived from platelets, and cMV not exposing PS (AV^–^) from platelets, endothelial cells, leukocytes and erythrocytes. In addition, cMV carrying thrombomodulin (CD141), both exposing AV or not, are also in lower concentrations in successfully aged octogenarians. This might be important on the grounds that, besides playing an essential role in coagulation, thrombomodulin has been shown to participate in the regulation of inflammatory processes (Conway, 2012). Consistent with these results, subjects who suffered a stroke show elevated concentrations of cMV from platelets, endothelial cells, erythrocytes and leukocytes with a prothrombotic phenotype at the long term (Chiva-Blanch et al., 2016). Patients with Alzheimer disease and cognitive decline show increased levels of neural-derived MV of specific phenotypes (Kapogiannis et al., 2019; Eren et al., 2020), which are able to identify Alzheimer-related cognitive decline again at the long term. Additionally, we have previously observed in a Chilean cohort that frail older adults present elevated concentrations of total, AV^+^ and AV^–^ cMV derived from monocytes, natural killers and platelets (Arauna et al., 2020), which contributes explaining, at least in part, the increased risk of CVD in these subjects. In the same line, in another subset of the OCTABAIX cohort, subjects with previous CVD and cognitive decline presented higher concentrations of the antifibrinolytic proteins alpha-2-antiplasmin (A2AP) and coagulation factor XIII-B-chain (FXIIIB) compared to non-CVD subjects with preserved cognition and functionality (Cubedo et al., 2015). This was accompanied with a coordinated imbalance in several proteins related to inflammation and coagulation (Cubedo et al., 2017), pinpointing the association between cytokine-triggered inflammation and vascular microthrombosis in unhealthy aging, with CVD and cognitive impairment as underlying causes. In this setting, it is plausible, therefore, that elevated concentrations of cMV from platelets and endothelial cells with both AV^+^ and AV^–^ phenotypes contribute to the pathological state associated with non-successful aging (cognitive impairment, CVD and diminished quality of life).

In our study subjects, malnutrition scores were associated with high platelet MV release and low immune cell-derived MV blebbing, suggesting that malnutrition might be associated with platelet hyperreactivity and immune dysfunction. However, data on dietary intake and physical activity was not available, and therefore this association requires further research.

The study of the effects of (non-successful) aging is primarily focused on tissues and cells, and the effects of aging in circulating factors is largely unexplored. After multivariate adjustments, and although B coefficients are low, we have observed that octogenarians successfully aged also show lower concentrations of cMV from platelets and endothelial cells non-exposing phosphatidylserine in their surface, thus with lower potential prothrombotic effect. This observation fits the inflammaging theory (Santoro et al., 2020), which attributes to subclinical and chronic low-grade inflammation, that may be perpetuated by sustained elevated concentrations of proinflammatory cMV, an underlying cause of aging. In fact, senescent cells have been shown to have a proinflammatory secretome (Eitan et al., 2017; Takasugi, 2018) with increased release of MV and overall extracellular vesicles (Willis et al., 2020) with proinflammatory phenotypes (Alibhai et al., 2020; Salminen et al., 2020), contributing to age-related comorbidities (Misawa et al., 2020). However, these studies have been performed in *in vitro* or in *in vivo* models of aging (Russell et al., 2020; Salminen et al., 2020), and human data is lacking.

In this study, successful aging has been defined as good functional (Barthel Index > 90 points, and Lawton Index scores ≤ 7 for women and ≤4 men) and cognitive status (MEC > 24 points) and no need for institutionalization (Formiga et al., 2011). However, this definition of successful aging has been criticized (Laplante, 2014), because wellbeing and perception of successful aging may not be limited to elders with full functionality and independence (Golja et al., 2020), or “healthy” from a strictly biomedical/clinical perspective, and may not consider the individual feeling, social aspects (Ding et al., 2020), depression, quality of life (Jang, 2020), or the “disability paradox” (Albrecht and Devlieger, 1999). It is of course more likely that disabled elders do not report successful aging, yet still some elders with some degrees of disability may report wellness (Mitra et al., 2020), especially those enclosed in aging services systems facilitating their risk reduction of chronic diseases associated with aging and promoting social inclusion. In this setting, successful aging may be better defined and understood including qualities and capacities appreciated in the elderly.

This study is not exempt of limitations. It is a cross-sectional study of associative nature and therefore hypothesis-generating. Further studies are needed to evaluate the causality or consequence of elevated concentrations of cMV in functional and cognitive performance. In addition, data on age of CVD onset, and data on other comorbidities such as neurodegenerative diseases or cancer are lacking. However, it is unlikely that subjects with advanced Alzheimer disease or with a tumoral process, for instance, or within the acute phase of a major adverse cardiovascular event are not being institutionalized. Samples used in this study belong to a biobank, and have been carefully stored under −80∘C for 10 years. This might provoke an unknown impact on MV stability. However, samples were carefully processed. They were collected and immediately frozen in aliquots in 2009, and were processed and analyzed within 3 months in 2019. All samples were handled following the same procedure and all samples were frozen and thawed only for one specific analysis, in this case the MV analysis. Therefore, the potential effects of long-term effects of storage at −80∘C in MV stability is comparable and homogeneous between samples. Finally, the lower limit of detection of the cytometer used for cMV quantification is between 0.2 and 0.3 μm. Therefore, information on extracellular vesicles from smaller sizes are missing and might be of relevance.

In summary, our study shows an inverse association between endothelial- and platelet-derived cMV release at the bloodstream and higher scores in functional and cognitive tests reflecting successful aging. Our results contribute in the accumulating evidence of the link between unsuccessful aging, cognitive decline and vascular inflammation and damage, determined here as elevated circulating levels of MV from platelets and endothelial cells at the bloodstream. To our knowledge and as previously mentioned, this is the first study associating low concentrations of platelet- and endothelial cell-derived cMV with successful aging, which opens new future lines of research, such as, for instance, the evaluation of the potential of cMV in adulthood as predictive biomarker of successful aging, from a holistic and comprehensive perspective, in the late elderly, or the identification of subjects requiring specific strategies to prevent vascular dysfunction and the onset of cognitive and functional decline, in order to modulate the rate of non-successful aging.

## Data Availability Statement

The raw data supporting the conclusions of this article will be made available by the authors, without undue reservation.

## Ethics Statement

This study involving human participants was reviewed and approved by CEIm of the Jordi Gol i Gurina Foundation. The patients/participants provided their written informed consent to participate in this study.

## Author Contributions

GC-B, TP, and LB: conceptualization. GC-B and AV-F: methodology. FF and AF: validation. GC-B: formal analysis. LB, FF, and AF: investigation, all, resources. TP: data curation. GC-B: writing—original draft preparation, writing—review and editing, all. LB: funding acquisition. All authors have read and agreed to the published version of the manuscript.

## Conflict of Interest

The authors declare that the research was conducted in the absence of any commercial or financial relationships that could be construed as a potential conflict of interest.

## Publisher’s Note

All claims expressed in this article are solely those of the authors and do not necessarily represent those of their affiliated organizations, or those of the publisher, the editors and the reviewers. Any product that may be evaluated in this article, or claim that may be made by its manufacturer, is not guaranteed or endorsed by the publisher.
