# Supplementary material for: Functional and Cognitive Decline Is Associated With Increased Endothelial Cell Inflammation and Platelet Activation: Liquid Biopsy of Microvesicles in Community- Dwelling Octogenarians
Source: Front Cell Dev Biol. 2021 Jul 29;9:716435. doi: 10.3389/fcell.2021.716435 (PMC8358681; doi:10.3389/fcell.2021.716435)
Supplement: Supplementary file 1 [file Data_Sheet_1.pdf]

Supplementary Material

1 Supplementary Data

cMV Isolation and Quantification by Flow Cytometry

Two hundred seventy-five microliters of frozen plasma aliquots were thawed on melting ice for 1 hour and centrifuged at 1300×g, 10 minutes, at room temperature to guarantee complete cell removal. Then, 250μL of plasma were transferred to another vial and centrifuged at 20 000×g for 30 minutes at room temperature to pellet circulating microvesicles (cMV). The supernatants were discarded, and the cMV-enriched pellet was washed once with citrate-PBS solution before a second equal centrifugation step was made. Finally, the cMV pellets were resuspended in 100μL citrate-PBS. Afterwards, 5μL of washed cMV suspensions were diluted in 30μL PBS buffer containing 2.5 mmol/L $CaCl_2$ (annexin V -AV- binding buffer). Thereafter, combinations of 5μL of CF405M-conjugated AV, which has high affinity for phosphatidylserine, with 2 specific monoclonal antibodies (1–5μL each; Supplementary Table 1) labeled with fluorescein isothiocyanate (FITC) or phycoerythrin (PE), or the isotype-matched control antibodies were added in a final volume of 50μL annexin binding buffer to label and characterize phosphatidylserine -positive (AV^+) cMV with bioactive and biomarker molecules from their parental cells, according to Supplementary Table 1 and Supplementary Figure 2 and 3. Samples were incubated 20 minutes at room temperature in the dark and diluted with annexin V binding buffer before being immediately analyzed on a FACSCanto II flow cytometer.

Acquisition was performed at 1 minute per sample and flow rate (FR) was measured before each experiment. Forward scatter, side scatter and fluorescence data were obtained with the settings in the logarithmic scale. The lower detection limit was placed as a threshold above the electronic noise of the SSC and FSC. To identify positive marked events, thresholds were also set within the second logarithm of fluorescence, based on samples incubated with the same final concentration of isotype-matched control antibodies after titration experiments, and checked in single stained controls at the beginning of the study (Supplementary Figure 2).

cMV were identified and quantified based on their forward scatter/side scatter characteristics according to their size, defined as 200-1000µm, the lower limit of detection of our cytometer according to the Megamix-Plus FSC beads for cytometer settings in microvesicle (microparticle) analysis (Supplementary Figure 2). cMV were also classified as AV positive or negative (AV^+ or AV^-), and therefore, to phosphatidylserine externalization, and reactivity to cell-specific monoclonal antibodies (Supplementary Figure 3). Appropriate controls (annexin V binding buffer only, binding buffer with antibodies and isotypes, and unstained MV in binding buffer) were run every day and representative plots are shown in Supplementary Figure 2. In addition, one sample was treated with the detergent saponin at 5%, to provoke MV lysis, thus indirectly demonstrating MV integrity. Data were analyzed with FACSDiva software (BD). The cMV concentration (number of cMV per µL of plasma) was determined according to Nieuwland's formula, where: Number of MV/µL plasma = N × [100/5] × [500/FR] × [1/250].

All buffers were prepared on the same day and filtered through 0.2µm pore size filters under vacuum to reduce background noise.

2 Supplementary Figures and Tables

2.1 Supplementary Figure 1

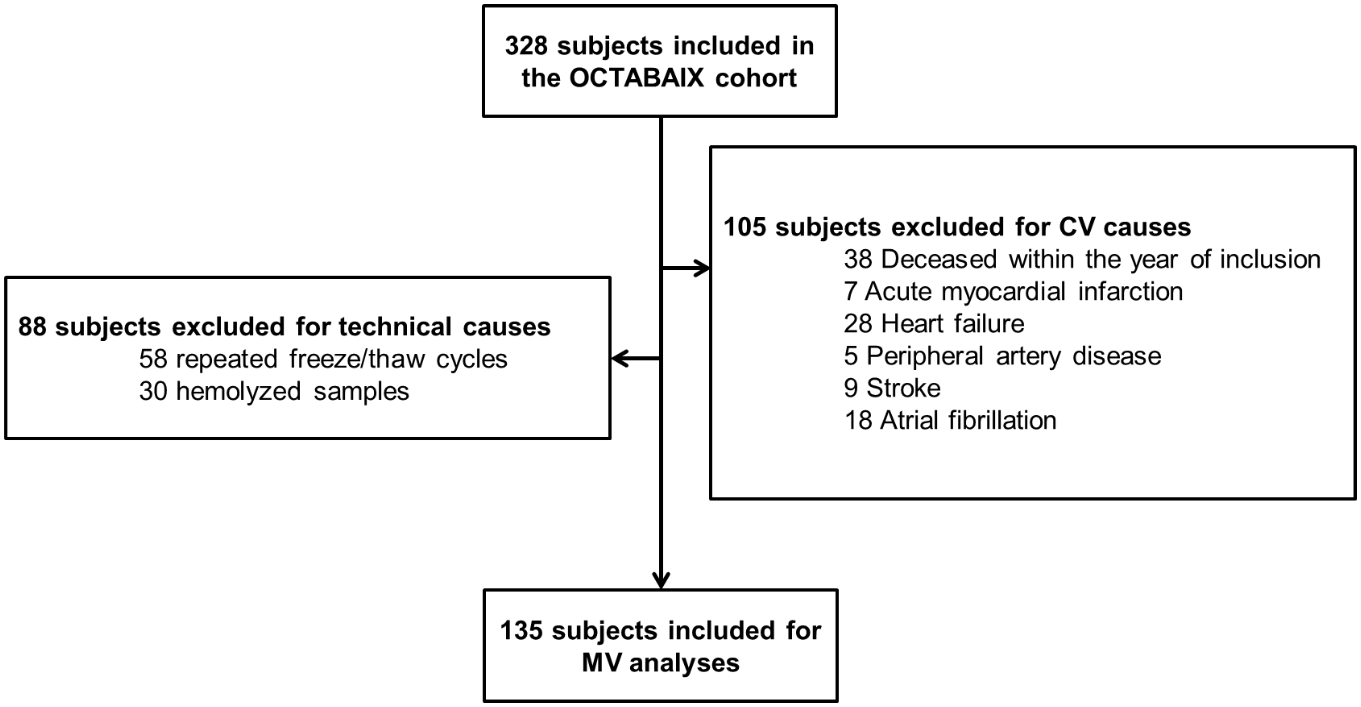

Supplementary Figure 1. Flowchart of the selection of patients for the study. Exclusion criteria in this study was having a CV event within the year before the inclusion in the study (n=105), repeated freeze/thaw cycles (n=58) and hemolyzed samples (n=30). Therefore, 135 subjects were included for microvesicle (MV) analyses.

2.2 Supplementary Figure 2

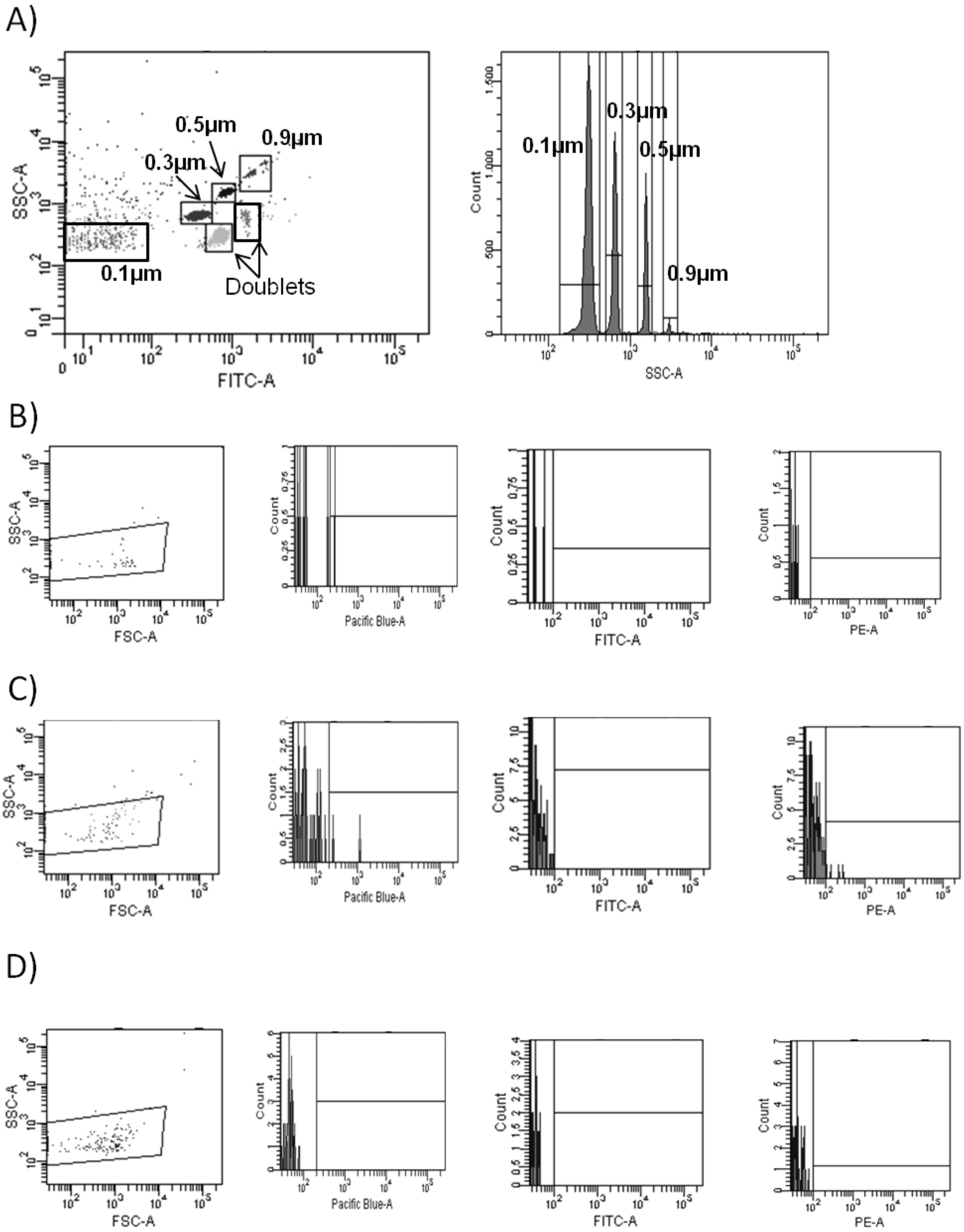

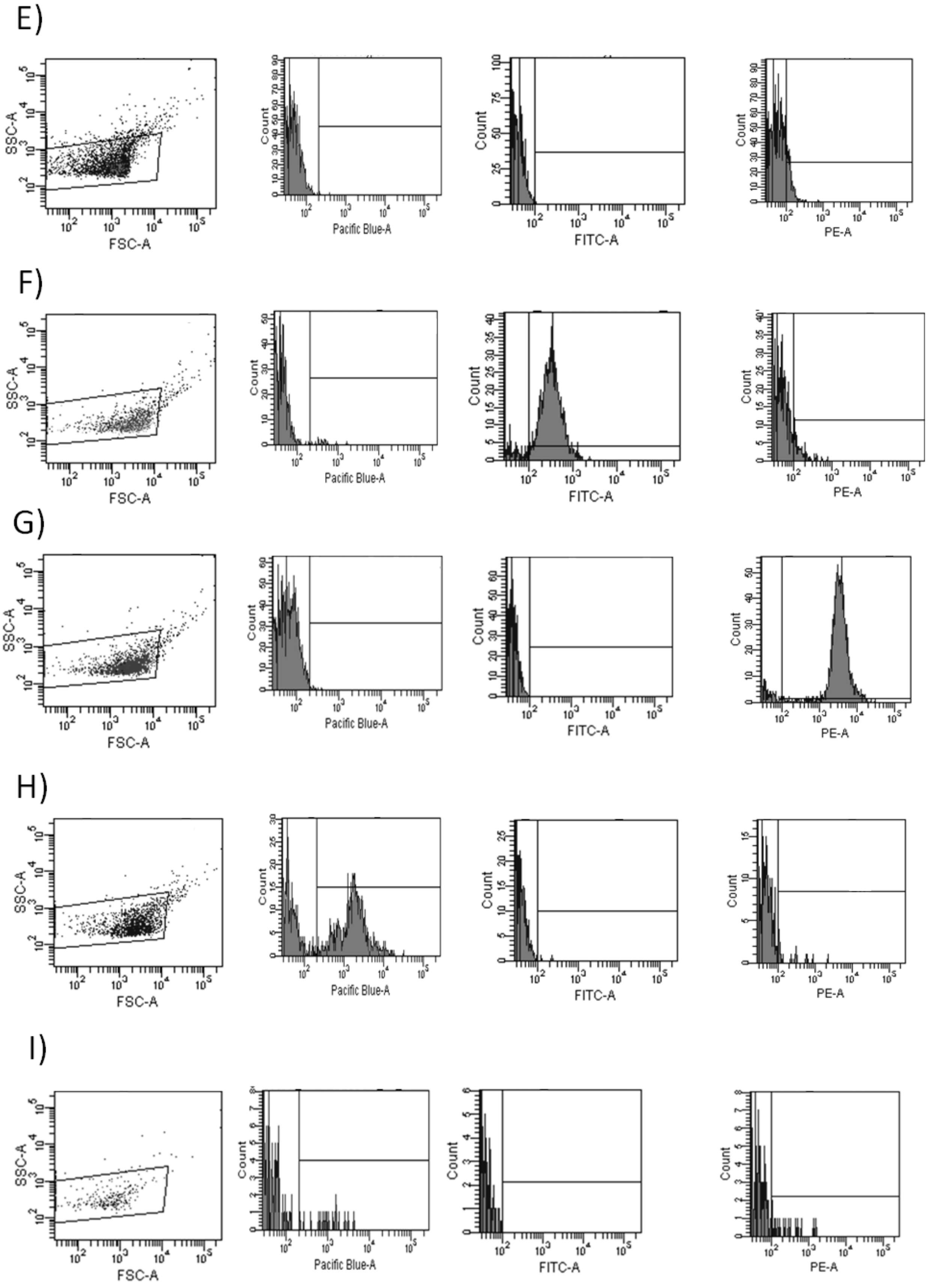
E)
F)
G)
H)
I)
SSC-A
FSC-A
Count
Pacific Blue-A
FITC-A
PE-A

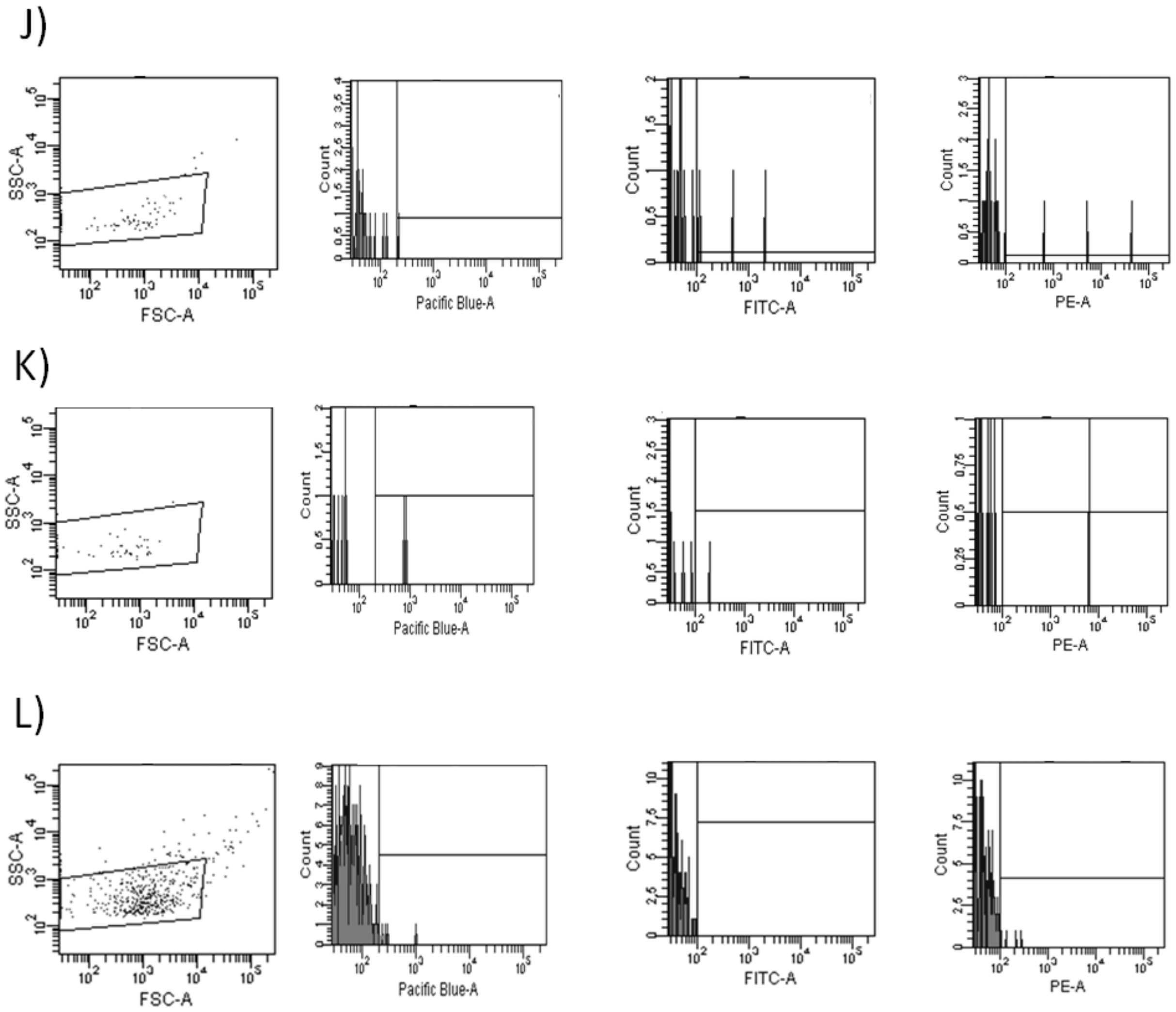

Supplementary Figure 2. Flow cytometry controls for MV analysis. Representative FSC/SSC dot plots and CF405M (in the Pacific Blue channel), FITC and PE histograms of: A) Megamix-Plus FSC beads; B) Annexin V binding buffer (ABB) only; C) ABB with FITC-, PE-labeled antibodies and AV- CF405M; D) Unstained MV; E) FITC- and PE-labeled isotype controls; F) FITC- stained sample; G) PE- stained sample; H) CF405M - stained sample; I) Dilution of the sample 1/4; J) Dilution of the sample 1/10; K) Dilution of the sample 1/50; and L) Detergent-treated MV sample (saponin 5% in ABB).

2.3 Supplementary Figure 3

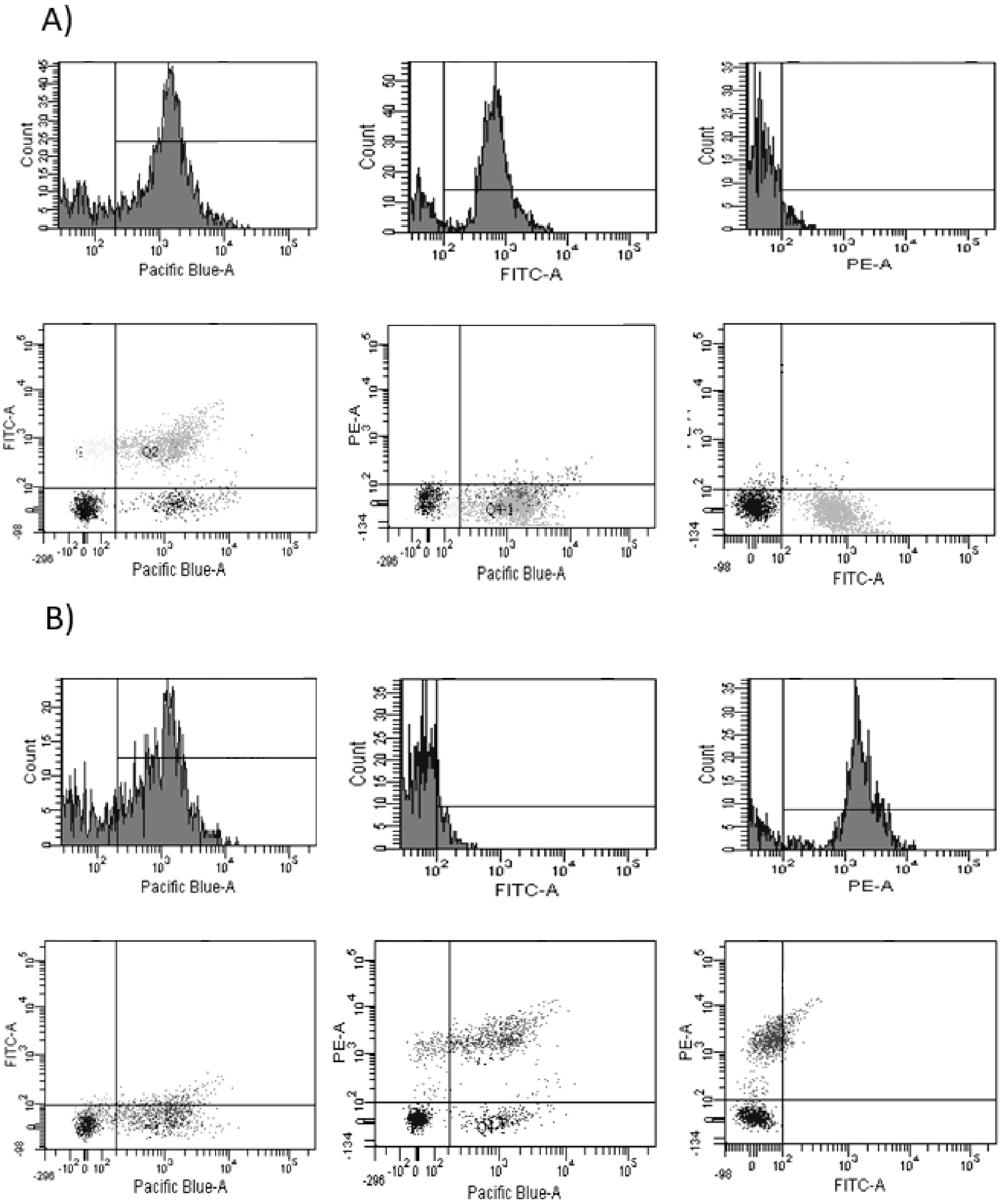

C)

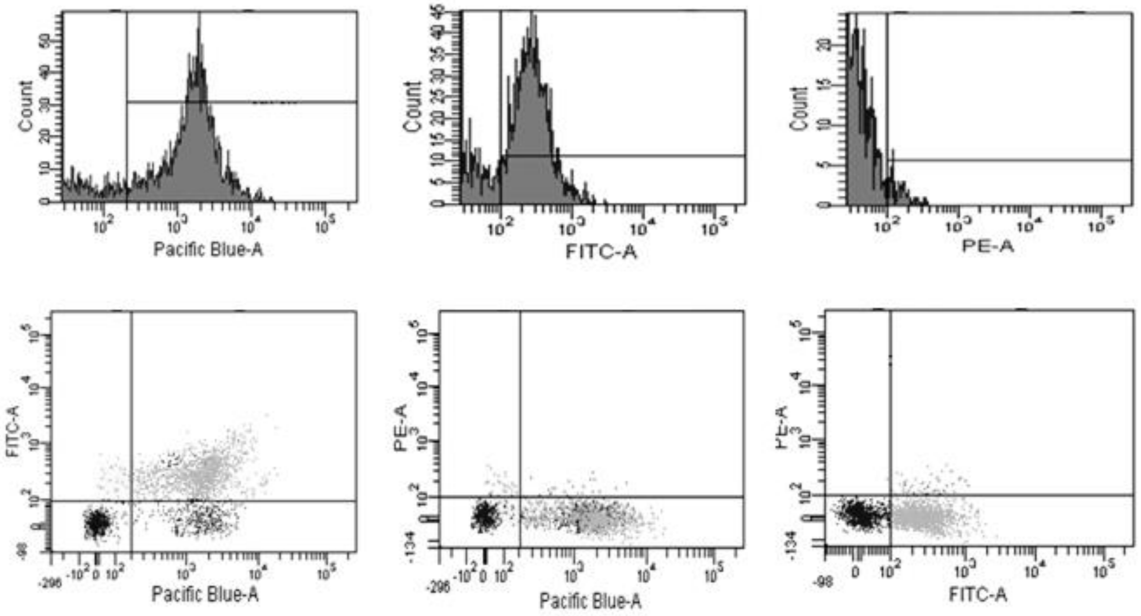

Supplementary Figure 3. Representative histograms and dot plots of: A) Microvesicles (MV) stained with CD41-CD61-FITC/CD141-PE/AV-CF405M; B) MV stained with CD31-FITC/CD41a-PE/AV-CF405M; and C) MV stained with CD36-FITC/GPVI-PE/AV-CF405M. AV denotes annexin V. CF405M is quantified in the Pacific Blue channel.

2.4 Supplementary Figure 4

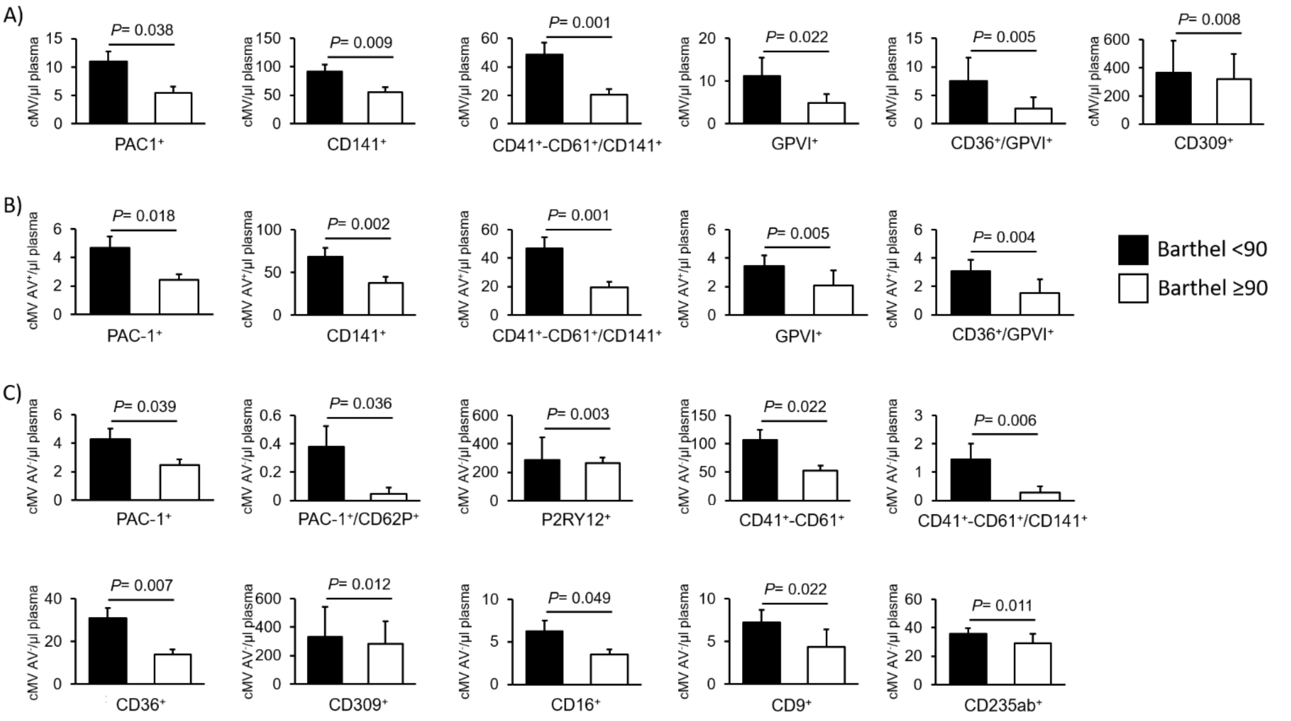

Supplementary Figure 4. cMV levels according to Barthel Index Score in the 135 octogenarians included in the study. Results are shown as mean+SEM of concentrations of: A) total microvesicles; B) AV^+ microvesicles; and C) AV^- cMV. Sixty-eight subjects had a Barthel index score <90 (in black) and 66 ≥90 (in white). AV indicates annexin V and cMV, circulating microvesicles. Used markers for MV phenotyping are shown in Supplementary Table 1.

2.5 Supplementary Figure 5

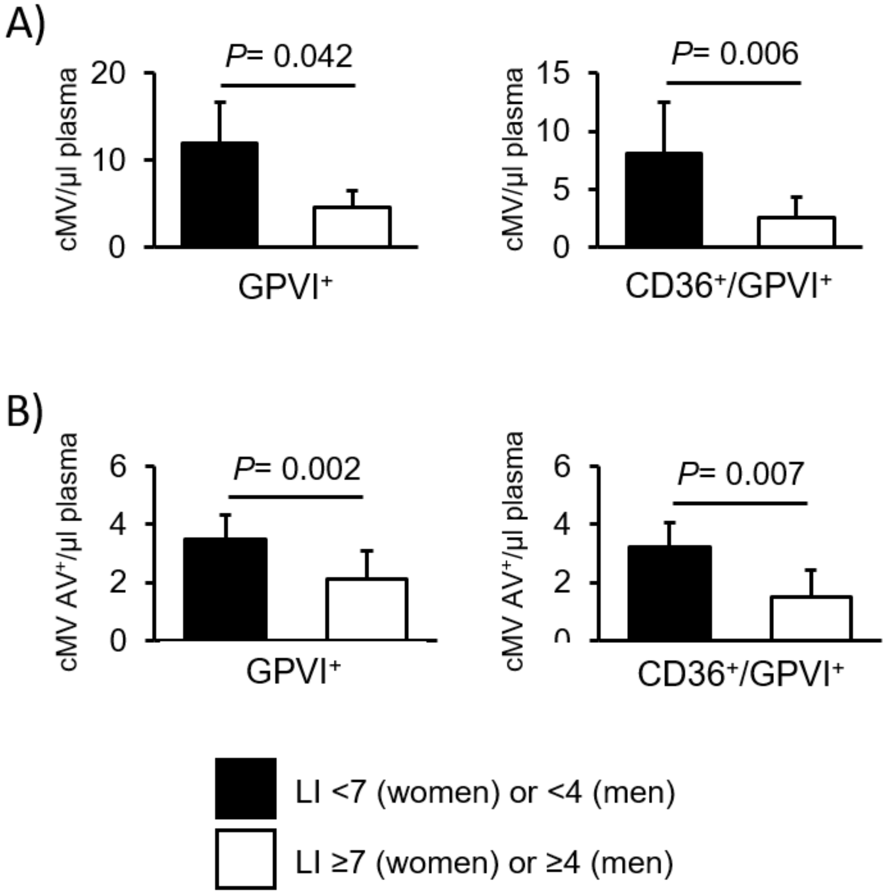

Supplementary Figure 5. cMV levels according to Lawton Index Score in the 135 octogenarians included in the study. Results are shown as mean+SEM of concentrations of: A) total microvesicles; and B) AV^{+} microvesicles. Sixty-six subjects had a Lawton index score <7 (form women) or <4 (for men), and 69 ≥7 or 4 for women and men, respectively. AV indicates annexin V and cMV, circulating microvesicles. Used markers for MV phenotyping are shown in Supplementary Table 1.

2.6 Supplementary Figure 6

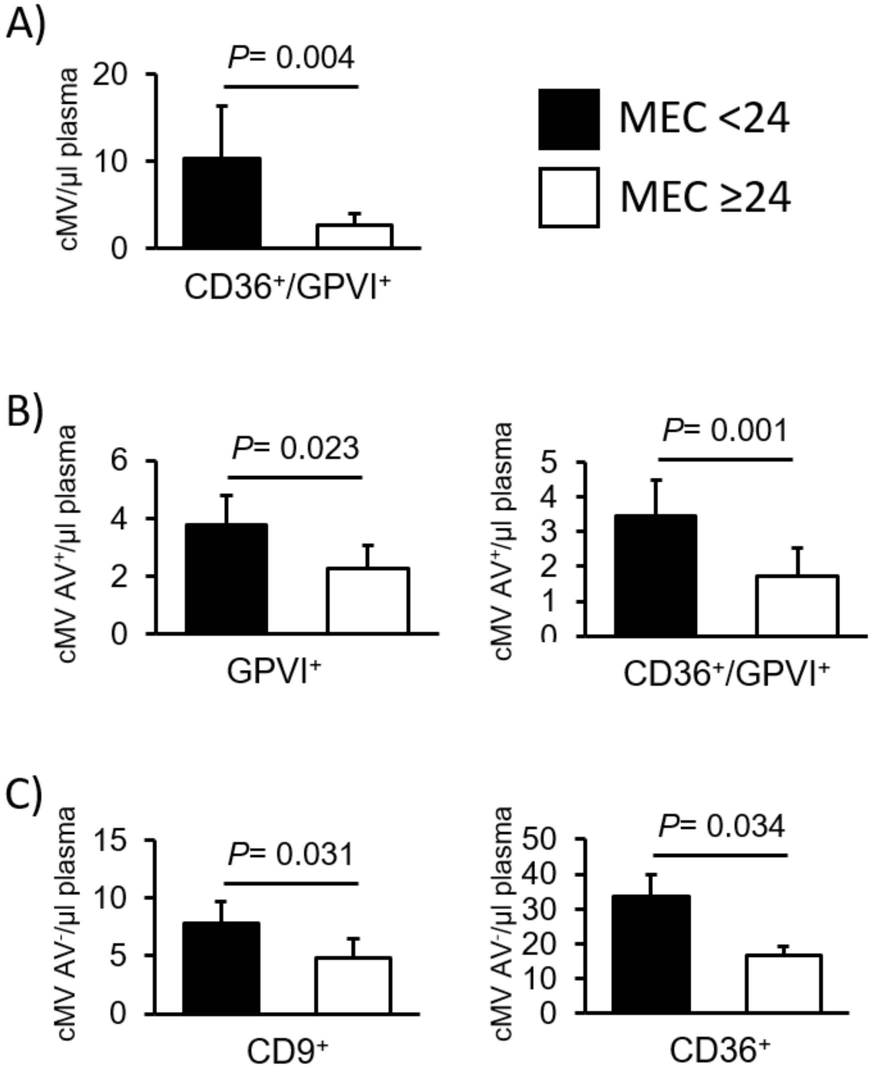

Supplementary Figure 6. cMV levels according to MEC Score in the 135 octogenarians included in the study. Results are shown as mean+SEM of concentrations of: A) total microvesicles; B) AV^+ microvesicles; and C) AV^- microvesicles. Eighty-eight subjects had a MEC score ≥24 MEC score and 47 had a MEC score <24. AV indicates annexin V and cMV, circulating microvesicles. Used markers for MV phenotyping are shown in Supplementary Table 1.

2.7 Supplementary Figure 7

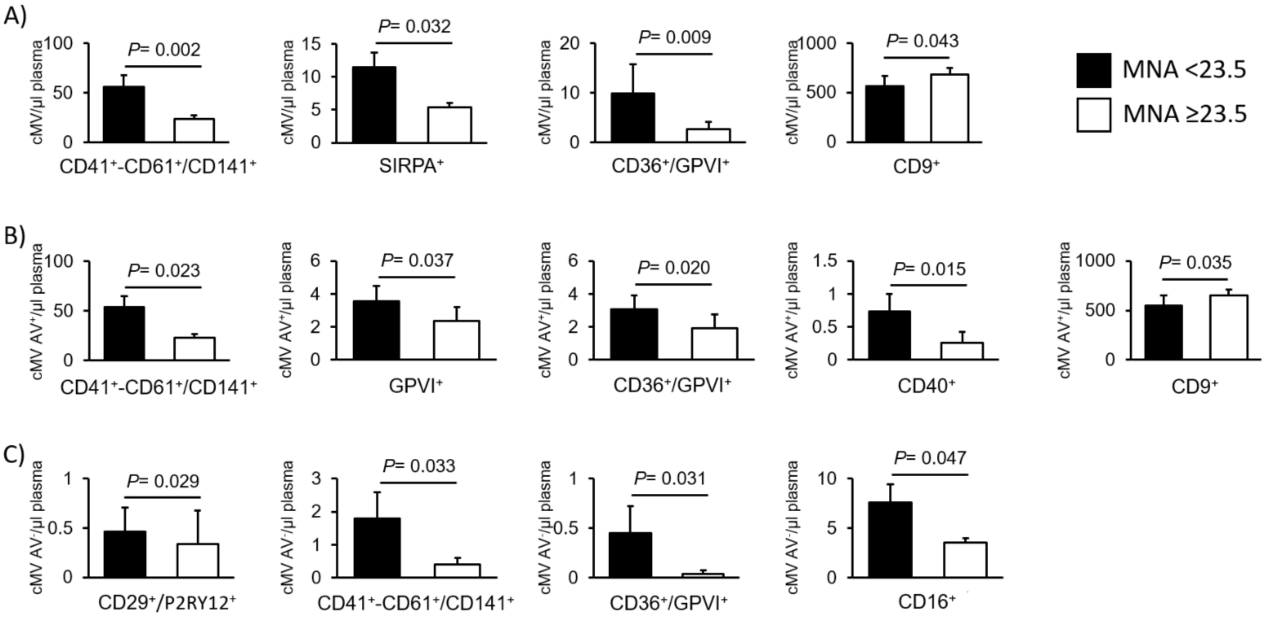

Supplementary Figure 7. cMV levels according to MNA Score in the 135 octogenarians included in the study. Results are shown as mean+SEM of concentrations of: A) total microvesicles; B) AV$^+$ microvesicles; and C) AV$^-$ microvesicles. Eighty-seven subjects had a MNA score ≥23.5 MEC score and 48 had a MNA score <23.5. AV indicates annexin V and cMV, circulating microvesicles. Used markers for MV phenotyping are shown in Supplementary Table 1.

2.8 Supplementary Table 1. Antibodies used for circulating microvesicle phenotyping.

Antibody	Alternative name	Expression
AV	phosphatidylserine-binding protein	Widely expressed
CD142	Tissue Factor	Widely expressed
CD9	Tetraspanin	Widely expressed
CD29	Integrin beta-1	Widely expressed
CD40	Tumor necrosis factor receptor superfamily member 5	Widely expressed
CD309	Vascular endothelial growth factor receptor 2	Endothelial cells
CD146	Melanoma Cell Adhesion Molecule	Endothelial cells
CD62E	E-Selectin	Endothelial cells
CD41a	Integrin αIIb or Platelet GPIIb	Platelets
CD62P	P-Selectin	Platelets
CD41-CD61	Integrin alpha IIb beta 3	Platelets
CD61	β_3-integrin	Platelets
P2RY12	G-protein coupled Purinergic receptor	Platelets
PAC-1	$\alpha_{IIb}\beta_3$-integrin	Platelets
GPVI	Glycoprotein VI	Platelets
CD31	Platelet endothelial cell adhesion molecule (PECAM)-1	Leukocytes, platelets and endothelial cells
CD36	platelet glycoprotein 4 (GPIV)	Platelets, erythrocytes, and monocytes
CD141	Thrombomodulin	Monocytes, neutrophils, platelets, endothelial and dendritic cells
CD11b	macrophage-1 antigen (Mac-1)	Leukocytes
CD66	Carcino-Embryonic Antigen-like Cellular Adhesion Molecules -1, -3, -5 and -6	Granulocytes
CD15	Sialyl Lewis X antigen	Granulocytes and monocytes
CD14	Lipopolysaccharide receptor	Monocytes and macrophages

CD16	Fcγ Receptor III	Natural killer, neutrophils, monocytes and macrophages
CD56	Neural cell adhesion molecule (NCAM)	Natural killers and neural cells
SIRPA	Signal regulatory protein α	Myeloid cells
CD34	Mucosialin	Progenitor and stem cells
Desmin	Muscle-specific type III intermediate filament	Cardiomyocytes
CX43	Connexin 43	Widely expressed
CD235a	Glycoporin A	Erythrocytes

2.9 Supplementary Table 2. cMV levels according to successful aging in the 135 octogenarians included in the study.

MV (number/µL plasma)	Successful aging (n=48)	Non-successful aging (n=87) First degree (n= 28)	Second degree (n= 23)	Third degree (n= 36)	*P*[1]	*P*[2]
$AV^{+/-}$	4987.89 ± 4997.32	5090.73 ± 4266.23	3170.07 ± 3625.09	3870.75 ± 2676.23	0.567	0.115
$CD146^{+}$	8.58 ± 10.48	12.14 ± 17.57	16.53 ± 23.1	13.57 ± 20.84	0.732	0.851
$CD62E^{+}$	278.61 ± 263.41	324.53 ± 417.25	347.31 ± 463.2	342.08 ± 634.75	0.604	0.885
$CD146^{+}/CD62E^{+}$	7.15 ± 7.23	11.36 ± 17.11	12.87 ± 17.02	9.06 ± 13.99	0.936	0.751
$CD31^{+}$	343.5 ± 434.18	338.98 ± 534.63	359.11 ± 483.71	374.32 ± 295.78	0.677	0.248
$CD41a^{+}$	1732.96 ± 1640.12	1554.02 ± 1891.9	1562.03 ± 1607.01	1858.04 ± 1377.2	0.531	0.188
$CD31^{+}/CD41a^{+}$	334.06 ± 430.95	324.47 ± 495.74	355.69 ± 483.07	371.55 ± 296.21	0.707	0.232
$CD31^{+}/CD41a^{-}$	1.51 ± 1.95	10.19 ± 38.91	3.1 ± 3.58	2.77 ± 4.01	0.044	0.134
$PAC1^{+}$	6.26 ± 10.04	5.52 ± 8.54	12.03 ± 15.08	10.68 ± 14.57	0.304	0.112
$CD62P^{+}$	302.7 ± 284.29	268.77 ± 272.1	261.21 ± 340.44	449.36 ± 505.49	0.552	0.332
$PAC1^{+}/CD62P^{+}$	0.91 ± 2.91	0.19 ± 0.69	1.91 ± 4.22	0.87 ± 2.21	0.823	0.162
$CD142^{+}$	137.5 ± 116.25	112.03 ± 102.16	147.93 ± 96.33	140.37 ± 124.21	0.905	0.441
$CD14^{+}$	29.06 ± 38.64	18.05 ± 19.12	37.82 ± 47.64	34.31 ± 49.99	0.546	0.669
$CD142^{+}/CD14^{+}$	16.22 ± 36.17	10.79 ± 17.6	16.73 ± 29.59	19.17 ± 37.93	0.891	0.973
$CD142^{+}/CD41a^{+}$	42.68 ± 90.75	32.23 ± 39.05	51.43 ± 66.03	61.82 ± 84.76	0.529	0.579
$CD309^{+}$	257.46 ± 1210	80.55 ± 260.03	839.29 ± 2609.22	347.76 ± 1875.95	0.425	0.505
$CD61^{+}$	2404.58 ± 4022.53	1462.09 ± 1440.97	3337.97 ± 6758.09	2794.78 ± 5691.85	0.349	0.256
$CD29^{+}$	764.53 ± 807.54	607.28 ± 591.56	718.22 ± 841.91	926.76 ± 890.45	0.619	0.334
$P2RY12^{+}$	863.07 ± 840.98	687.61 ± 1110.05	949.23 ± 1759.3	960.57 ± 1949.55	0.032	0.125
$CD29^{+}/P2RY12^{+}$	236.77 ± 321.64	140.8 ± 217.14	187.36 ± 339.1	219.21 ± 264.62	0.078	0.107
$CD41^{+}$-$CD61^{+}$	1638.9 ± 1581.71	1281.78 ± 1138.02	1362.24 ± 1331.53	1606.98 ± 1404.55	0.464	0.609
$CD141^{+}$	51.72 ± 70.53	64.17 ± 67.02	92.4 ± 96.24	98.82 ± 106.43	0.033	0.104
$CD141^{+}/CD41^{+}$-$CD61^{+}$	19.46 ± 30.49	27.93 ± 33.87	43.38 ± 52.32	55.16 ± 80.06	0.009	0.039
$CD56^{+}$	20.74 ± 25.43	15.73 ± 19.1	13.83 ± 16.67	16.1 ± 16.66	0.429	0.679

$CD34^+$	142.26 ± 140.5	132.56 ± 119.08	191.1 ± 245.23	230.38 ± 397.16	0.728	0.882
$CD56^+/CD34^+$	6.06 ± 11.8	4.66 ± 5.31	6.12 ± 6.49	4 ± 3.87	0.490	0.711
$CD16^+$	47.12 ± 140.97	27.42 ± 30.47	38.72 ± 42.67	56.15 ± 72.09	0.181	0.506
$CD14^+/CD16^+$	25.49 ± 122.85	8.87 ± 25.89	13.46 ± 33	9.42 ± 26.89	0.510	0.792
$CD14^+/CD16^-$	32.21 ± 122.08	14.37 ± 18.38	20.37 ± 27.35	16.81 ± 24.28	0.626	0.553
$CD66^+$	36.07 ± 174.72	7.46 ± 13.48	8.46 ± 14.2	6.19 ± 6.71	0.191	0.560
$CD15^+$	76.07 ± 275.03	27.99 ± 42.74	49.92 ± 66.55	51.06 ± 73.05	0.250	0.362
CD66+/CD15+	14.12 ± 63.54	4.13 ± 13.25	5.12 ± 14.44	2.13 ± 5.26	0.844	0.776
$CD66^+/CD15^-$	21.92 ± 118.38	3.2 ± 2.85	3.42 ± 3.83	3.82 ± 4.64	0.152	0.549
$SIRPa^+$	6.8 ± 9.37	5.24 ± 6.1	7.98 ± 11.76	9.93 ± 14.25	0.594	0.788
$SIRPa^+/CD34^+$	2.06 ± 4.01	2.43 ± 3.9	3.69 ± 4.9	3.2 ± 4.79	0.081	0.223
$SIRPa^+/CD34^-$	4.33 ± 5.88	2.81 ± 3.84	2.25 ± 3.24	4.37 ± 5.83	0.426	0.429
$CD40^+$	0.74 ± 3.52	1.87 ± 3.01	1.25 ± 2.22	1.87 ± 1.42	0.004	0.117
$CD9^+$	682.49 ± 695.92	621.04 ± 664.62	604.14 ± 577.79	616.88 ± 686.21	0.291	0.720
$CD40^+/CD9^+$	0.11 ± 0.76	0.6 ± 2.79	0.12 ± 0.56	0.08 ± 0.46	0.583	0.929
$CD3^+$	14.56 ± 10.35	11.33 ± 12.17	12.15 ± 11.93	9.89 ± 10.33	0.119	0.281
$CD45^+$	357.24 ± 516.22	370.22 ± 573.57	210.18 ± 214.67	227.94 ± 222.42	0.092	0.078
$CD3^+/CD45^+$	8.37 ± 7.7	6.09 ± 8.53	4.89 ± 9.2	4.59 ± 6.73	0.085	0.137
$Desmin^+$	429.66 ± 1954	48.4 ± 143.86	290.48 ± 618.07	290.74 ± 687.82	0.303	0.115
$CX43^+$	120.75 ± 681.36	10.1 ± 18.84	53.17 ± 154.09	54.54 ± 221.63	0.100	0.146
$Desmin^+/CX43^+$	16.98 ± 100.04	0.68 ± 2.13	4.19 ± 10.84	1.93 ± 4.93	0.452	0.426
$CD235ab^+$	454.03 ± 559.62	376.49 ± 283.19	348.05 ± 280.06	443.23 ± 335.33	0.898	0.601
$CD11b^+$	112.66 ± 313.47	78.54 ± 68.32	134.76 ± 207.3	96.61 ± 187.36	0.119	0.080
$CD36^+$	1201.44 ± 1222.08	927.24 ± 788.86	1051.68 ± 1158.2	1334.21 ± 1454.58	0.341	0.751
$GPVI^+$	5.79 ± 18	2.22 ± 3.12	5.36 ± 8.54	18.46 ± 46.87	0.186	0.048
$CD36^+/GPVI^+$	0.52 ± 1.98	0.91 ± 2.09	2.74 ± 5.51	2.78 ± 10.76	0.024	0.776
AV^+ MV						
AV^+	1845.83 ± 1291.72	1783.99 ± 1664.22	1639.91 ± 1404.56	1874.2 ± 1291.29	0.386	0.512
$CD146^+$	0.98 ± 4.66	0.01 ± 0.01	0.66 ± 1.67	0.75 ± 1.63	0.432	0.117
$CD62E^+$	96.99 ± 72.33	81.59 ± 75.69	121.41 ± 178.12	111.09 ± 116.08	0.392	0.747

$CD146^+/CD62E^+$	0.5 ± 1.56	0.22 ± 1.07	0.28 ± 0.83	0.51 ± 1.38	1.000	0.711
$CD31^+$	310.99 ± 409.33	268.3 ± 289.45	277.37 ± 372.05	352.04 ± 270.62	0.638	0.120
$CD41a^+$	1466.44 ± 1428.64	1347.41 ± 1446.03	1422.53 ± 1546.96	1676.96 ± 1204.34	0.745	0.181
$CD31^+/CD41a^+$	308.02 ± 410.31	263.25 ± 279.25	275.94 ± 371.56	351.44 ± 270.69	0.683	0.117
$CD31^+/CD41a^-$	0.67 ± 1.39	1.16 ± 2.45	0.75 ± 1.47	0.6 ± 1.29	0.797	0.904
$PAC1^+$	2.79 ± 3.47	2.62 ± 5.04	2.49 ± 5.86	5.89 ± 6.86	0.899	0.040
$CD62P^+$	255.78 ± 255.83	262.97 ± 269.72	249.13 ± 330.86	289.18 ± 337.02	0.675	0.811
$PAC1^+/CD62P^+$	0.29 ± 0.83	0.01 ± 0.01	0.15 ± 0.62	0.01 ± 0.01	0.068	0.090
$CD142^+$	37 ± 64.89	32.56 ± 46.97	61.48 ± 68.16	58.42 ± 73.61	0.512	0.387
$CD14^+$	13.86 ± 25.93	12.43 ± 19.81	11.14 ± 17.11	10.06 ± 15.43	0.295	0.721
$CD142^+/CD14^+$	10.2 ± 25.89	8.25 ± 17.34	5.24 ± 10.63	9.99 ± 32.94	0.564	0.495
$CD142^+/CD41a^+$	36 ± 88.44	29.9 ± 37.92	40.24 ± 53.84	50.82 ± 69.34	0.514	0.572
$CD309^+$	29.69 ± 112.44	16.3 ± 48.8	64.94 ± 191.16	25.69 ± 106.51	0.132	0.222
$CD61^+$	1425.67 ± 1123.33	1315.86 ± 1405.98	1216.14 ± 1430.52	1639.6 ± 1305	0.350	0.142
$CD29^+$	761.41 ± 773.08	594.65 ± 587.4	694.63 ± 817.49	826.36 ± 756.46	0.466	0.342
$P2RY12^+$	551.8 ± 569.91	328.43 ± 481.75	683.23 ± 1539.29	520.13 ± 574.74	0.062	0.069
$CD29^+/P2RY12^+$	186.46 ± 214.24	140.7 ± 217.18	95.74 ± 151.1	216.66 ± 261.76	0.052	0.098
$CD41^+$-$CD61^+$	1545.79 ± 1490.04	1228.42 ± 1116.86	1283.26 ± 1244.9	1376.58 ± 1190.76	0.321	0.682
$CD141^+$	35.26 ± 57.96	44.37 ± 48.94	75.14 ± 92.76	71.05 ± 86.42	0.006	0.031
$CD141^+/CD41^+$-$CD61^+$	18.67 ± 29.71	27.02 ± 33.27	41.15 ± 50.69	52.76 ± 78.49	0.009	0.041
$CD56^+$	11.52 ± 18.65	7.18 ± 9.41	6.9 ± 7.16	6.93 ± 6.02	0.534	0.840
$CD34^+$	109.5 ± 127.96	92.77 ± 94.92	119.03 ± 145.71	136.49 ± 133.35	0.951	0.714
$CD56^+/CD34^+$	4.54 ± 9.94	4.12 ± 5.99	3.62 ± 3.84	3.01 ± 3.17	0.473	0.845
$CD16^+$	40.13 ± 137.01	26.6 ± 32.06	31.81 ± 39.18	40.3 ± 55.32	0.177	0.571
$CD14^+/CD16^+$	21.37 ± 122.54	8.74 ± 25.53	5.24 ± 8.25	3.28 ± 7.53	0.922	0.556
$CD14^+/CD16^-$	23.59 ± 105.82	9.83 ± 14.94	11.48 ± 17.75	10.19 ± 16.55	0.687	0.818
$CD66^+$	5.97 ± 11.46	6.31 ± 13.47	3.12 ± 4.03	3.59 ± 5.96	0.680	0.943
$CD15^+$	24.81 ± 30.6	19.82 ± 28.76	35.27 ± 54.66	27.93 ± 42.13	0.211	0.558
CD66+/CD15+	2.07 ± 6.25	3.25 ± 12.53	1.87 ± 3.23	0.74 ± 1.79	0.673	0.716
$CD66^+/CD15^-$	2.17 ± 4.89	1.36 ± 1.83	1.14 ± 2.06	1.39 ± 2.41	0.753	0.909

$SIRPa^+$	2.62 ± 4.57	2.62 ± 5.8	2.5 ± 5.47	5.05 ± 8.63	0.943	0.747
$SIRPa^+/CD34^+$	1.34 ± 2.78	1.5 ± 3.53	2.03 ± 4.12	2.82 ± 4.78	0.219	0.247
$SIRPa^+/CD34^-$	0.85 ± 1.84	1.01 ± 2.78	1.44 ± 6.45	0.51 ± 1.69	0.122	0.264
$CD40^+$	0.06 ± 0.38	0.94 ± 2.82	0.71 ± 1.84	0.34 ± 1.31	0.056	0.140
$CD9^+$	626.24 ± 616.37	616.74 ± 661.36	597.64 ± 572.24	598.58 ± 669.29	0.390	0.808
$CD40^+/CD9^+$	0.01 ± 0.01	0.55 ± 2.68	0.12 ± 0.56	0.01 ± 0.01	0.268	0.313
$CD3^+$	4.05 ± 14.25	3.28 ± 6.72	3.62 ± 3.75	3.74 ± 5.8	0.544	0.560
$CD45^+$	385.19 ± 294.69	344.22 ± 541.62	154.77 ± 167.09	316.94 ± 239.35	0.100	0.078
$CD3^+/CD45^+$	1.98 ± 7.87	1.07 ± 2.55	0.95 ± 1.72	0.93 ± 2.13	0.821	0.962
$Desmin^+$	84.24 ± 270.58	34.08 ± 100.13	84.48 ± 168.68	98.65 ± 153.78	0.813	0.206
$CX43^+$	14.27 ± 59.7	4.85 ± 8.47	8.49 ± 14.33	8.5 ± 11.19	0.467	0.414
$Desmin^+/CX43^+$	3.23 ± 19.99	0.29 ± 1.11	2.62 ± 7.06	0.9 ± 3.92	0.418	0.517
$CD235ab^+$	413.04 ± 510.64	346.29 ± 278.04	303.04 ± 254.73	411.33 ± 366.87	0.880	0.737
$CD11b^+$	59.95 ± 156.09	44.86 ± 46.24	63.94 ± 54.11	49.78 ± 83.03	0.085	0.057
$CD36^+$	1168.1 ± 1178.28	914.42 ± 779.88	1028.78 ± 1135.8	1271.82 ± 1373.67	0.342	0.779
$GPVI^+$	2.56 ± 9.27	1.11 ± 1.99	2.38 ± 5.17	4.76 ± 7.42	0.050	0.014
$CD36^+/GPVI^+$	1.89 ± 8.83	0.6 ± 1.54	2.26 ± 5.13	4.4 ± 7.73	0.045	0.004
AV^- MV						
AV^-	2048.48 ± 2622.23	2254.24 ± 3165.55	4250.48 ± 7484.85	3023 ± 1187.59	0.974	0.385
$CD146^+$	7.15 ± 9.26	10.68 ± 17.11	15.39 ± 22.26	10.88 ± 17.39	0.652	0.788
$CD62E^+$	163.8 ± 205.5	184.49 ± 240.6	225.9 ± 376.29	290.6 ± 696.31	0.660	0.675
$CD146^+/CD62E^+$	6.08 ± 6.8	10.48 ± 17	12.03 ± 16.81	10.48 ± 16.88	0.634	0.832
$CD31^+$	6.43 ± 10.04	70.68 ± 330.48	7.15 ± 11.76	11.84 ± 15.54	0.114	0.233
$CD41a^+$	81.49 ± 85.15	206.61 ± 754.97	79.95 ± 97.03	124.53 ± 134.15	0.717	0.444
$CD31^+/CD41a^+$	5.73 ± 11.71	61.22 ± 294.33	5.48 ± 11.55	10.53 ± 18.7	0.611	0.722
$CD31^+/CD41a^-$	0.84 ± 1.65	9.03 ± 37.03	1.67 ± 2.63	2.11 ± 3.7	0.028	0.093
$PAC1^+$	2.62 ± 3.66	2.9 ± 5.4	4.89 ± 4.53	3.77 ± 6.09	0.274	0.063
$CD62P^+$	8.97 ± 11.77	5.8 ± 7.6	12.08 ± 16.46	13.43 ± 19.19	0.715	0.676
$PAC1^+/CD62P^+$	0.06 ± 0.41	0.1 ± 0.5	0.12 ± 0.56	0.61 ± 1.49	0.179	0.040
$CD142^+$	80.85 ± 56.63	65.06 ± 60.12	75.9 ± 56.13	67.54 ± 49.73	0.180	0.355

$CD14^+$	11.66 ± 12.47	10.3 ± 14.96	16.18 ± 19.83	13.18 ± 22.36	0.537	0.456
$CD142^+/CD14^+$	5.35 ± 9.51	3.2 ± 4.32	3.87 ± 5.05	3.93 ± 5.03	0.487	0.856
$CD142^+/CD41a^+$	2.98 ± 5.84	1.31 ± 1.7	2.03 ± 2.55	2.27 ± 3.38	0.868	0.904
$CD309^+$	226.71 ± 1096.88	57.67 ± 215.28	737.88 ± 2370.42	317.05 ± 1772.63	0.598	0.093
$CD61^+$	973.12 ± 4101.83	146.23 ± 230.92	2121.83 ± 6813.31	1138.93 ± 5726.57	0.858	0.989
$CD29^+$	20.24 ± 44.98	12.64 ± 14.81	23.59 ± 35.59	34.12 ± 58.05	0.590	0.478
$P2RY12^+$	261.23 ± 281.81	180.77 ± 343.09	167.04 ± 294.44	433.81 ± 1741.2	0.896	0.673
$CD29^+/P2RY12^+$	0.66 ± 4.14	0.01 ± 0.01	0.71 ± 2.01	0.09 ± 0.49	0.525	0.102
$CD41^+$-$CD61^+$	59.69 ± 82.8	53.36 ± 72.37	78.98 ± 111.16	132.96 ± 167.91	0.204	0.295
$CD141^+$	14.69 ± 20.92	12.16 ± 17.58	18.61 ± 21.78	16.53 ± 22.52	0.493	0.680
$CD141^+/CD41^+$-$CD61^+$	0.01 ± 0.01	0.87 ± 2.72	2.12 ± 6.87	1.23 ± 3.11	0.004	0.026
$CD56^+$	6.1 ± 7.63	5.54 ± 8.59	9.04 ± 15.81	7.76 ± 11.14	0.687	0.458
$CD34^+$	27.82 ± 34.2	39.79 ± 41.25	124.39 ± 236.55	89 ± 362.64	0.066	0.280
$CD56^+/CD34^+$	0.76 ± 1.44	0.78 ± 1.6	1.87 ± 3.53	0.97 ± 2.09	0.684	0.755
$CD16^+$	3.99 ± 5.12	3.49 ± 4.24	6.91 ± 12.96	5.95 ± 9	0.958	0.921
$CD14^+/CD16^+$	1.04 ± 1.6	0.58 ± 1.33	0.36 ± 0.92	0.98 ± 1.85	0.124	0.251
$CD14^+/CD16^-$	8.25 ± 19.91	5.15 ± 5.61	5.36 ± 8.71	6.09 ± 14.51	0.905	0.498
$CD66^+$	3.36 ± 3.11	2.52 ± 3.29	2.51 ± 3.01	1.97 ± 2.58	0.057	0.173
$CD15^+$	4.57 ± 25.56	8.97 ± 15.87	7.29 ± 7.94	5.74 ± 8.47	0.122	0.316
CD66+/CD15+	0.81 ± 5.39	0.1 ± 0.51	0.01 ± 0.01	0.01 ± 0.01	0.100	0.320
$CD66^+/CD15^-$	1.57 ± 10.49	1.84 ± 2.5	2.28 ± 2.99	1.75 ± 2.51	0.159	0.164
$SIRPa^+$	4.18 ± 7.2	2.62 ± 3.35	3.69 ± 4.18	2.93 ± 3.91	0.844	0.821
$SIRPa^+/CD34^+$	0.73 ± 2.82	0.94 ± 2.39	1.6 ± 3.14	1.16 ± 3.78	0.243	0.167
$SIRPa^+/CD34^-$	3.46 ± 5.33	1.69 ± 2.69	2.85 ± 4.6	3 ± 4.5	0.451	0.526
$CD40^+$	0.71 ± 3.09	0.75 ± 1.57	0.46 ± 1.02	0.62 ± 1.3	0.278	0.703
$CD9^+$	4.81 ± 18.5	4.17 ± 6.87	6.5 ± 10.69	8.13 ± 13.04	0.129	0.225
$CD40^+/CD9^+$	0.11 ± 0.76	0.01 ± 0.01	0.01 ± 0.01	0.08 ± 0.45	0.686	0.731
$CD3^+$	10.76 ± 8.8	8.05 ± 10.96	7.18 ± 9.05	5.99 ± 8.45	0.072	0.124
$CD45^+$	33.84 ± 33.17	22.96 ± 24.21	35.91 ± 70.08	39.02 ± 65.91	0.129	0.493
$CD3^+/CD45^+$	1.53 ± 7.12	2.62 ± 3.93	0.79 ± 1.92	1.75 ± 3.51	0.143	0.315

Desmin$^+$	20.28 ± 28.94	5.82 ± 11.43	29.75 ± 45.79	24.28 ± 28.46	0.349	0.184
CX43$^+$	5.79 ± 6.51	3.13 ± 4.51	4.46 ± 5.65	3.69 ± 4.89	0.070	0.143
Desmin$^+$/CX43$^+$	0.19 ± 0.69	0.2 ± 0.71	0.66 ± 1.44	0.41 ± 1.17	0.347	0.435
CD235ab$^+$	30.26 ± 57.47	30.24 ± 23.29	33.45 ± 30.56	37.23 ± 31.07	0.063	0.084
CD11b$^+$	49.58 ± 195.17	34.08 ± 66.73	74.47 ± 180.39	35.24 ± 119.97	0.676	0.061
CD36$^+$	15.19 ± 21.15	14.04 ± 19.37	22.91 ± 26.4	39.32 ± 47.89	0.029	0.003
GPVI$^+$	3.04 ± 9.06	1.03 ± 2.18	2.85 ± 5.36	3.71 ± 7.26	0.783	0.486
CD36$^+$/GPVI$^+$	0.06 ± 0.4	0.01 ± 0.01	0.25 ± 0.79	0.5 ± 2.1	0.424	0.291

Results are expressed as mean ± sd. P^1 for the comparison between successful and non-successful aging (Mann-Whitney test), and P^2, for the comparison between successful aging and different degrees of non-successful aging (Kruskal-Wallis test). Successful aging was defined as good functional (Barthel Index >90 points, and Lawton Index scores >7 for women and >4 men) and cognitive status (MEC >24 points), and no need for institutionalization. Non-successful aging was further categorized in having one (first degree), two (second degree) or three scores (third degree) below good functional and/or cognitive status (Barthel Index ≤90 points, Lawton Index scores ≤7 for women and ≤4 men, and/or MEC ≤24 points). AV indicates annexin V and cMV, circulating microvesicles. Used markers for MV phenotyping are shown in Supplementary Table 1.
